# Supplementary material for: Relationship between general practice capitation funding and the quality of primary care in England: a cross-sectional, 3-year study
Source: BMJ Open. 2019 Nov 7;9(11):e030624. doi: 10.1136/bmjopen-2019-030624 (PMC6858150; doi:10.1136/bmjopen-2019-030624)
Supplement: Author's manuscript [file bmjopen-2019-030624.draft_revisions.pdf]

BMJ Open

BMJ Open is committed to open peer review. As part of this commitment we make the peer review history of every article we publish publicly available.

When an article is published we post the peer reviewers' comments and the authors' responses online. We also post the versions of the paper that were used during peer review. These are the versions that the peer review comments apply to.

The versions of the paper that follow are the versions that were submitted during the peer review process. They are not the versions of record or the final published versions. They should not be cited or distributed as the published version of this manuscript.

BMJ Open is an open access journal and the full, final, typeset and author-corrected version of record of the manuscript is available on our site with no access controls, subscription charges or pay-per-view fees (http://bmjopen.bmj.com).

If you have any questions on BMJ Open's open peer review process please email info.bmjopen@bmj.com

BMJ Open

The relationship between general practice funding and the quality of primary care in England

Journal:	*BMJ Open*
Manuscript ID	bmjopen-2019-030624
Article Type:	Research
Date Submitted by the Author:	22-Mar-2019
Complete List of Authors:	L'Esperance, Veline; King's College London, UK, School of Population Health and Environmental Sciences<br>Gravelle, Hugh; University of York, Centre for Health Economcs<br>Schofield, Peter; King's College London, UK, School of Population Health and Environmental Sciences<br>Santos, Rita; University of York, Centre for Health Economics<br>Asthworth, Mark; King's College London, UK, School of Population Health and Environmental Sciences
Keywords:	PRIMARY CARE, Quality in health care < HEALTH SERVICES ADMINISTRATION & MANAGEMENT, HEALTH ECONOMICS, Health inequalities

SCHOLARONE™
Manuscripts

Title:

The relationship between general practice funding and the quality of primary care in England

Authors:

Veline L'Esperance[1], Hugh Gravelle[2], Peter Schofield[3], Rita Santos[4], Mark Ashworth[5]

1. Doctoral Research Fellow, School of Population Health & Environmental Sciences, Kings College London, London, UK
2. Professor of Economics, Centre for Health Economics, University of York, York, UK
3. Wolfson Lecturer, School of Population Health & Environmental Sciences, Kings College London, London, UK
4. Research Fellow, Centre for Health Economics, University of York, York, UK
5. Reader in Primary Care, School of Population Health & Environmental Sciences, Kings College London, London, UK

Corresponding author email: mark.ashworth@kcl.ac.uk

Abstract:

Objective To explore the relationship between general practice funding and quality ratings based on general practice quality inspections.

Design Cross-sectional study pooling three years of primary care administrative data.

Setting UK primary care.

Participants 7310 practices (95% of all practices) in England which underwent Care Quality Commission (CQC) inspections between November 2014 and December 2017.

Main outcome measures CQC ratings. Ordered logistic regression methods were used to predict the relationship between practice capitation funding and CQC ratings in each of 5 domains of quality: caring, effective, responsive, safe and well-led, together with an overall practice rating.

Results Higher funding was significantly associated with higher CQC ratings across all five quality domains; caring (odds ratio [OR] 1.93, 95% confidence interval [CI]: 1.27 to 2.95); effective (OR 1.46, 95% CI: 1.01 to 2.12); responsive (OR 1.57, 95%CI : 1.10 to 2.24); safe

For peer review only - http://bmjopen.bmj.com/site/about/guidelines.xhtml

(OR 1.72, 95% CI: 1.24 to 2.38); well-led (OR 1.81, 95% CI: 1.38 to 2.54); and overall rating (OR 1.85, 95% CI: 1.36 to 2.50).

Conclusion: Higher capitation funding per patient was consistently associated with higher ratings across all CQC domains and in the overall practice rating. This study suggests that measured dimensions of the quality of care are related to the underlying funding allocated to each general practice, implying that additional funding may be associated with higher levels of primary care quality.

Strengths and limitations

- A cross-sectional study covering three years of primary care data
- The definition of primary care quality used in this study was multidimensional, based on inspection findings and covering patient safety, patient experience, clinical effectiveness.
- The association between the achievement of quality ratings and practice funding was explored, adjusted for known confounders
- Although based on a near complete sample of general practices in England, bias may have been introduced by data coding and recording errors
- Longer term and prospective studies are required to strengthen causal inferences

For peer review only - http://bmjopen.bmj.com/site/about/guidelines.xhtml

Introduction

Improving the quality of care is a major focus of UK government health policy(1). High quality health care has three main components: clinical achievement, patient experience and patient safety(2). There is wide variation between general practices in the achievement of clinical care quality indicators and patient reported satisfaction(3).

It is important to understand whether variations in the quality of care provided across practices are related to variations in their funding. Healthcare quality regulation of healthcare in England is currently undertaken by the Care Quality Commission, focuses on outcomes for patients and has a wide range of enforcement powers, including closure and deregistration of services, if essential standards are not met.(4)

Studies of the relationship between quality and funding in English general practices have largely focussed on the Quality and Outcomes Framework (QOF) which rewards practices for higher quality care, as defined by the achievement of clinical process and outcome targets. The QOF has had limited impact on reducing secondary care costs(5) or improving primary care performance(6, 7). In terms of financial incentivisation, the QOF accounted for approximately 7.8% of funding received by general practices in England in 2016(8). In contrast, capitation payments represent the largest proportion of funding to general practice (54% in 2016) and are related to the number of registered patients in each practice(8), adjusted for factors thought to increase the demand on primary care services(9). Other components of general practice funding include additional payments for postgraduate training, the provision of additional clinical services ('enhanced services') and various

For peer review only - http://bmjopen.bmj.com/site/about/guidelines.xhtml

reimbursements to cover the costs of premises, computers and for some practices, dispensing medication(10).

Greater capitation spending on general practices has been found to be associated with reductions in secondary care usage and costs, and increased patient satisfaction(11). Studies have also shown that leadership within the practice organisation plays a key role in the delivery of high quality care(12). Until recently, nationally derived metrics of measures of inspection-based primary care quality were unavailable. Since October 2014 all general practices have been subjected to inspections by the Care Quality Commission (CQC) (13), (4). The CQC reports on the extent to which practices are caring, effective, responsive to the needs of patients, safe, and well-led(4, 14) and also combines these five domains to produce an overall practice rating. In this study, we assess the relationship of practice capitation funding with overall CQC ratings and with the individual CQC domains.

Methods

Data sources

We linked practice-level data on NHS payments to general practice(15) to CQC inspection ratings(14), NHS administrative datasets, General and Personal Medical Services Statistics (16), and small area Census and socio-economic data from Neighbourhood Statistics(17).

Care Quality Commission Ratings

CQC ratings are based on publicly available data (such as QOF and General Practice Patient Survey(18)), practice inspections and interviews with patients and staff(14). We used CQC ratings for practices with completed CQC reports first inspected between November 2014 and

For peer review only - http://bmjopen.bmj.com/site/about/guidelines.xhtml

December 2017 (n = 7310, 95% of all practices). For practices which required reinspection only the first inspection score was included in the analysis. The five domains of quality described by CQC inspections are summarised in Table 1; each is rated on a 4-point scale.

Practice data

Data for all general practices in England were obtained from the General and Personal Medical Services database, for 2014/15, 2015/16 and 2016/17 financial years (16). Our use of practice based demographic data followed a previously used methodology(19). Patient characteristics included the proportion of patients aged 0 to 4 years, proportion of patients aged 75 years or older and proportion of nursing home patients. Deprivation data for each general practice was attributed as the mean of the Index of Multiple Deprivation (IMD) 2015(17) weighted by the proportion of practice patients resident in each Lower Layer Super Output area (LLSOA). Neighbourhood ethnicity (proportion Asian or black) derived from the 2011 national census, was attributed to practices weighted by the proportion of the practice population in each LLSOA(20). The following practice characteristics were included: region (North, Midlands, London and South), contract type (General Medical Services or Personal Medical Services), minimum distance from an acute hospital, dispensing status (whether the practice dispensed as well as prescribed medication), singlehanded practice status and training practice status. We did not include practice staffing (GPs, nurses, other staff) as explanatory variables in the model because staffing is likely to be directly affected by practice funding and so inclusion of these variable may lead to an underestimate of the full effect of funding. Moreover, a major change in the way in which staffing data was collected in 2015/16 also means that we would have had to drop a large number of observations.

For peer review only - http://bmjopen.bmj.com/site/about/guidelines.xhtml

Practice funding data

Practice funding data was available for the financial years 2014/15, 2015/16 and 2016/7(21). We defined practice funding as the capitation payment per patient for each financial year. Capitation payments are weighted to reflect factors affecting GP workload (age, gender, patients in nursing and residential homes, small area measures of morbidity), rurality and an index of local staff costs which affect the cost of providing services(9).

Sample

We linked inspected practices (n=7310) with funding data for their year of inspection. We excluded atypical practices with ≤750 registered patients (n=10) or ≤500 patients per FTE GP (n= 8) following a previously used method(22). Practices with recorded negative (n= 2) or zero funding (n = 52) were excluded. The final analysis sample consisted of 7238 practices.

Data Analysis

Analysis was at GP practice level. Since the CQC rating outcomes are ordered categories we used ordered logistic regression to model the relationship between funding and the practice CQC ratings(23). Separate models were estimated for each domain.

The key explanatory variable was funding per 100 patients. In addition to patient and practice characteristic covariates, the regression models included year effects to allow for inspection year and annual general practice funding uplifts. We accounted for local area effects by adjusting for clustering at Clinical Commissioning Group (CCG) level. Multicollinearity was tested for by calculating the Variance Inflation Factor (VIF) and variables with a value for VIF>5 were excluded. The proportional odds assumption of the ordered logit model was also tested(24). We report the odds ratio from the estimated models.

For peer review only - http://bmjopen.bmj.com/site/about/guidelines.xhtml

For peer review only

We also calculated the average marginal effects of funding on the predicted probabilities of achieving overall ratings of “outstanding” and “inadequate” for all practices. We also compare the predicted probabilities of an “outstanding” overall rating for training versus non-training practices, single-handed versus multi-handed practices, and rural versus urban practices. STATA 14 (StataCorp, College Station, TX) was used for all statistical analyses.

Patient involvement

Funding for this study included funding of a dedicated patient involvement group. Patients were involved in developing plans for the study design, approving the outcome measures and commenting on the potential impact of outcomes. A lay summary was also provided.

Results

Summary statistics for the main characteristics of the general practices are provided in Table 2. Mean funding per registered patient increased from £77.49 in 2014/5 to £83.17 in 2016/7 (Table 3).

The distribution of practice ratings across each quality domain is shown in Figure 1. A total of 79% (n = 5774) of practices achieved an overall rating of ‘Good’, while only 4% (294) achieved an overall rating of ‘Outstanding’. ‘Inadequate’ ratings varied across the domains, from 1% (caring domain) to 6% (safety domain), with an ‘Inadequate’ rating of 4% for overall performance.

Figure 2 shows the difference in capitation funding for practices with the lowest quality rating compared to those with the highest quality rating. In each domain, ‘Inadequate’ practices

For peer review only - http://bmjopen.bmj.com/site/about/guidelines.xhtml

received less capitation funding; this was significant for three domains (caring, safe, well-led) and for the overall practice rating.

Table 4 reports the odds ratios on funding per 100 patients estimated from four regression models of overall practice CQC rating. In the first model funding is the only explanatory variable and remaining models have successive additional explanatory variables: year effects, patient characteristics, and practice characteristics. The unadjusted model shows an association between higher capitation funding and higher overall CQC ratings with an odds ratio (OR) of 1.48 (95% confidence interval (CI): 1.14 to 1.92). Allowing for the year of inspection increased the OR slightly to 1.55 (95%CI: 1.19 to 2.01). Additional allowance for patient characteristics (OR 1.73; 95%CI: 1.32 to 2.26) and practice characteristics (OR 1.85; 95%CI: 1.36 to 2.50) further increased the odds ratio.

Table 5 reports odds ratios for all the explanatory variables in the overall practice quality rating model with all the additional explanatory variables. In addition to higher practice funding, rural practice and training practice status were significantly associated with higher overall practice ratings. Conversely, lower CQC overall practice rating were associated with higher levels of deprivation, the proportions of patients from black and Asian ethnicities, and singlehanded practice status.

The odds ratio on capitation funding per 100 patients from the full models for each CQC domain are shown in Table 6. Higher funding was significantly associated with higher CQC ratings across all five quality domains.

For peer review only - http://bmjopen.bmj.com/site/about/guidelines.xhtml

We used the results from the ordered logistic regression models with the full set of explanatory variables to calculate the probability of achieving an overall practice rating of 'Outstanding' or 'Inadequate'. Figure 3 shows the average predicted probability of achieving the practice rating for each funding level calculated using actual values of practice non-funding characteristics (year effects, patient characteristics & practice characteristics). Higher funding was associated with reduced probability of achieving an 'Inadequate' ratings and increased probability of an 'Outstanding' quality rating.

We also compared the probability of achieving an 'Outstanding' rating at different levels of GP funding for training versus non-training practices (Figure 4), for single-handed versus group practices (Figure 5), and for rural versus urban practices (Figure 6). At all levels of funding training practices have higher probabilities than non-training, group practices have higher probabilities than single handed practices, and rural practices have higher probabilities than urban practices. In all cases higher capitation funding is associated with higher probabilities of an 'Outstanding' rating.

Sensitivity Analyses

The Brant test(24) assesses the proportional odds assumption that the distance between each category is equivalent. Four of the variables included in our model (region, proportion of patients aged 0-4 years, contract type and single-hander status) did not meet the assumption of proportionality of the odds ratios. However, our variable of interest, capitation funding per patient, did not violate the proportional odds assumption. A partial proportional odds model estimated by generalised ordered logistic regression, yielded similar results to our main

For peer review only - http://bmjopen.bmj.com/site/about/guidelines.xhtml

model: higher funding was significantly associated with increase probability of achieving an 'Outstanding' rating (OR 1.93, 95% CI: 1.23 to 3.06).

Discussion

This study has demonstrated that higher capitation funding is associated with significantly higher overall practice quality ratings and ratings across all individual domains.

Practice characteristics such as training practice and group practice status were also associated with higher quality ratings, representing primary care structures which support higher quality of care. However, some factors related to the registered practice population, such as urban location, social deprivation and larger proportions of ethnic minority patients were negatively associated with the practice quality of care rating. Many of these factors are already known to be negatively associated with reported patient satisfaction (25) and QOF achievement (26). Including them in the model led to a stronger association of practice funding with practice quality rating. The likely reason for this is that practice capitation funding is positively correlated with patient characteristics which have negative effects on the quality rating. Thus including these patient characteristics in the model removes a source of bias from omitted variables which would otherwise tend to underestimate the positive association of funding with the quality rating.

Strengths and weaknesses of the study

For peer review only - http://bmjopen.bmj.com/site/about/guidelines.xhtml

This is the first study to explore the relationship between practice-level capitation funding and CQC ratings. We have been able to estimate the probability of achieving higher CQC ratings with additional funding. A variety of sensitivity analyses included in the methodology have confirmed the robustness of the ordered logistic regression modelling. These findings are based on a near complete sample of general practices across England. Using data linkages from a wide range of sources, and multilevel statistical models, this study has been able to demonstrate the independent effect of practice funding and practice characteristics on quality ratings, which might otherwise be confounded in single-level analyses.

However, there are several limitations. Routinely collected data are subject to coding and recording errors. As with all observational studies, significant associations, even if large, may not be causal. Although a wide range of potential confounders were included in the models, residual confounding cannot be excluded.

Comparison with existing literature

These findings add to those of a previous study which found that increased general practice funding was associated with reduced emergency hospital admissions and A&E attendances(11). Further, it has demonstrated that the current capitation funding formula may contribute to the persistence of the inverse care law with deprived areas experiencing lower quality of care, as defined by inspection ratings(27). Consistent with our study, others have found that GP practice funding correlate negatively with healthcare need predictors such as deprivation and non-white ethnicity(28). Previous studies have also demonstrated that greater GP workload may be associated with higher levels of social deprivation and with a higher proportion of Asian patients(29). Similarly, practices with a greater proportion of

For peer review only - http://bmjopen.bmj.com/site/about/guidelines.xhtml

ethnically diverse patients reported worse patient experience(30). Though rural practice were more likely to achieve an 'Outstanding' rating, consultation rates and rurality do not appear to be associated(29).

Implications for policy and practice

This work provides further evidence of the association between general practice funding and the quality of primary care. A causal association is plausible and supports the argument that increased quality and safety of patient care may be achieved through additional investment. The recently published NHS Long Term Plan(31) outlines proposals to offer substantial increases to capitation payment together with an emphasis on inter-practice cooperation through the formation of primary care networks. Both factors are likely to further influence the relationship between funding and the quality of primary care and will require further study. The NHS Long Term Plan also emphasises the importance of collaborations between the CQC and practices working in local areas implying collective responsibility for improving the quality of care in localities. Our findings suggest that revisions to the primary care capitation formula are necessary to ensure that more funding is provided in areas of high deprivation and ethnic minority populations reduce inequalities in the quality of care.

Unanswered questions and future research

Future research should extend these findings to subsequent 3-year cycles of quality inspection. A longitudinal approach to analysis is likely to provide more accurate inference of model parameters, better control for the influence of omitted variables and generate more accurate predictions of quality outcomes. Qualitative study is likely to provide insight into

For peer review only - http://bmjopen.bmj.com/site/about/guidelines.xhtml

mechanisms underlying the link between better funded practices and higher quality rating achievement.

Conclusion

Higher capitation funding per patient was consistently associated with higher overall and domain quality ratings yielded by CQC inspections. This study suggests that measured and inspected dimensions of the quality of care are related to the underlying funding allocated to each general practice, implying that additional funding may be associated with higher levels of primary care quality.

What is already known on this topic

- Few studies on the relationship between capitation funding and quality
- In England, the QOF has had limited impact on reducing healthcare costs and improving primary care performance

What this study adds

- Capitation funding is associated with increased quality ratings across all domains
- The NHS Long Term Plan calls for increased funding to primary care, therefore it important to understand how and where this funding should be directed
- Our findings imply that further primary care funding is particularly needed in areas of high deprivation and ethnic minority populations where funding is insufficient to support high quality of care.

For peer review only - http://bmjopen.bmj.com/site/about/guidelines.xhtml

Contributors: VL, HG, PS, RS and MA contributed to the idea and design of the study. VL and PS led on data analysis with statistical advice from HG, RS and MA. VL produced the first draft of the paper; all co-authors contributed and approved the final draft. VE is the guarantor. The corresponding author attests that all listed authors meet authorship criteria and that no others meeting the criteria have been omitted.

Funding: This work was supported by the National Institute for Health Research (NIHR) who funded a Doctoral Research Fellowship for VE (reference, DRF-2017-10-132) and for RS (reference, DRF-2014-07-055). HG and RS were funded by the UK NIHR Policy Research Programme (Policy Research Unit in the Economics of Health and Social Care Systems: Ref 103/0001). The views expressed are those of the authors and not necessarily those of the NHS, the National Institute for Health Research, the Department of Health and Social Care or its arm's length bodies or other UK government departments. The findings presented are independent from the funders who have had no role in study design, data collection, data analysis, data interpretation, or writing of the report.

Competing interests: All authors have completed the ICMJE uniform disclosure form at www.icmje.org/coi_disclosure.pdf and declare: no support from any organisation for the submitted work; no financial relationships with any organisations that might have an interest in the submitted work in the previous three years; no other relationships or activities that could appear to have influenced the submitted work.

Ethical approval: Ethical approval not required for the use of aggregate practice level data as included in this study.

Dissemination declaration: the results have been disseminated to the patient involvement group involved in study design and commenting on potential impact of outcomes.

Data sharing: No additional data available.

For peer review only - http://bmjopen.bmj.com/site/about/guidelines.xhtml

Figures and Tables

Table 1: The five key domains for CQC Inspections

Domain	Description
**Safe**	Patients are protected from abuse and avoidable harm
**Effective**	Care, treatment and support achieves good outcomes, helps patients to maintain quality of life and is based on the best available evidence
**Caring**	Staff involve and treat patients with compassion, kindness, dignity and respect
**Responsive**	Services are organised so that they meet patients' needs
**Well-led**	The leadership, management and governance of the organisation make sure it's providing high-quality care that's based around the individual needs, that it encourages learning and innovation, and that it promotes an open and fair culture

Adapted from: CQC. The five key questions we ask(32)

Table 2. Characteristics of general practices and their populations in England

Variable	Mean	(5th, 95th centiles)
Patient adjusted Index of Multiple Deprivation, 2015	24.5	8.2, 46.1
Proportion of patients aged 0 to 4 years (%)	5.9	3.7, 8.8
Proportion of patients aged 75 years or older (%)	7.7	2.6, 12.9
Proportion of patients: nursing home residents (%)	0.5	0, 1.4
Proportion of patients: Asian or black ethnicity (%)	13.1	0.1, 53.1
List size per full-time equivalent (FTE) GP	1950	1066, 3315
List size per FTE non-clinical employed staff	703	392, 1103
List size per FTE nurse	7166	2810, 15507
Minimum distance of practice from acute hospital (km)	3.8	0.4, 11.8
Proportion of practices by rurality (%)		
Urban	85.5	
Hamlet, village, town & fringe	14.5	
Proportion of practices by region (%)		
North	30.3	
Midlands	29.4	
London	18.0	
South	22.3	
Proportion of practices by contract type (%)		
General Medical Services	59.4	
Personal Medical Services	40.6	
Proportion of dispensing practices (%)	14.6	
Proportion of singlehanded practices (%)	13.1	
Proportion of training practices (%)	30.4	

For peer review only

For peer review only - http://bmjopen.bmj.com/site/about/guidelines.xhtml

Table 3: Capitation funding per registered patient for inspected practices

Inspection year	N	Mean	(5th, 95th centiles)
2014/15	2232	77.49	59.54, 99.99
2015/16	3790	80.86	66.57, 101.66
2016/17	1148	83.71	67.74, 106.76

Table 4: Association of general practice funding (per 100 patients) with overall practice CQC rating: unadjusted and adjusted regression models

	Unadjusted	Year effects [1]	Patient characteristics [2]	Practice characteristics [3]
**Odds Ratio**	1.58**	1.55**	1.73***	1.85***
**95% CI**	1.19, 2.00	1.19, 2.01	1.32, 2.26	1.36, 2.50
**Observations**	7,168	7,168	7,144	7,045

1. Model has mean practice funding and year effects
2. As above + patient adjusted deprivation, proportion of patients aged 0 to 4 years, proportion of patients aged ≥75 years, proportion patients black or Asian ethnicity, proportion of nursing home residents
3. As above + region, minimum distance to hospital, contract type, dispensing status, training practice status, singlehanded

*p< 0.05; **p< 0.01; ***p< 0.001

For peer review only - http://bmjopen.bmj.com/site/about/guidelines.xhtml

Table 5: Association of general practice funding (with overall practice CQC rating: predictor variable in fully adjusted model.

Characteristics	Odds Ratio	95% CI
Capitation funding (per 100 patients)	1.85***	1.36, 2.50
Year 2	0.92	0.80, 1.05
Year 3	0.76**	0.64, 0.91
Deprivation	0.99**	0.98, 0.99
Patients aged 0 to 4 years (proportion)	1.00	0.95, 1.05
Patients aged 75 years or old (proportion)	0.99	0.96, 1.17
Patients in nursing home (proportion)	1.13	1.02, 1.26
Patients Asian or black ethnicity (proportion)	0.99*	0.99, 1.00
Region: Midlands§	0.64***	0.55, 0.76
Region: London§	0.56***	0.93, 0.98
Region: South§	0.48**	0.40, 0.58
Minimum distance to hospital	1.00	1.00, 1.00
Rurality (Yes/No)	1.50**	1.18, 1.92
Contract type (GMS/PMS)	1.08	0.96, 1.23
Dispensing Practice status (yes/no)	1.10	0.88, 1.38
Singlehanded practice (yes/no)	0.53***	0.44, 0.63
Training Practice status (yes/no)	2.30***	1.99, 2.65
***p< 0.05; **p< 0.01; ***p< 0.001**		

§ Comparator Region: North

For peer review only

Table 6: Ordered logistic models: effect of general practice funding on each CQC domain rating

Domains	Odds Ratio	95% CI
Caring	1.93**	1.27, 2.95
Effective	1.46*	1.01, 2.12
Responsive	1.57*	1.10, 2.24
Safe	1.72*	1.24, 2.38
Well-led	1.87***	1.38, 2.54
Overall	1.85***	1.36, 2.50

Adjusted for year effects, patient characteristics & practice characteristics &
*p< 0.05; **p< 0.01; ***p< 0.001

Figure 1: Distribution of CQC ratings by each domain

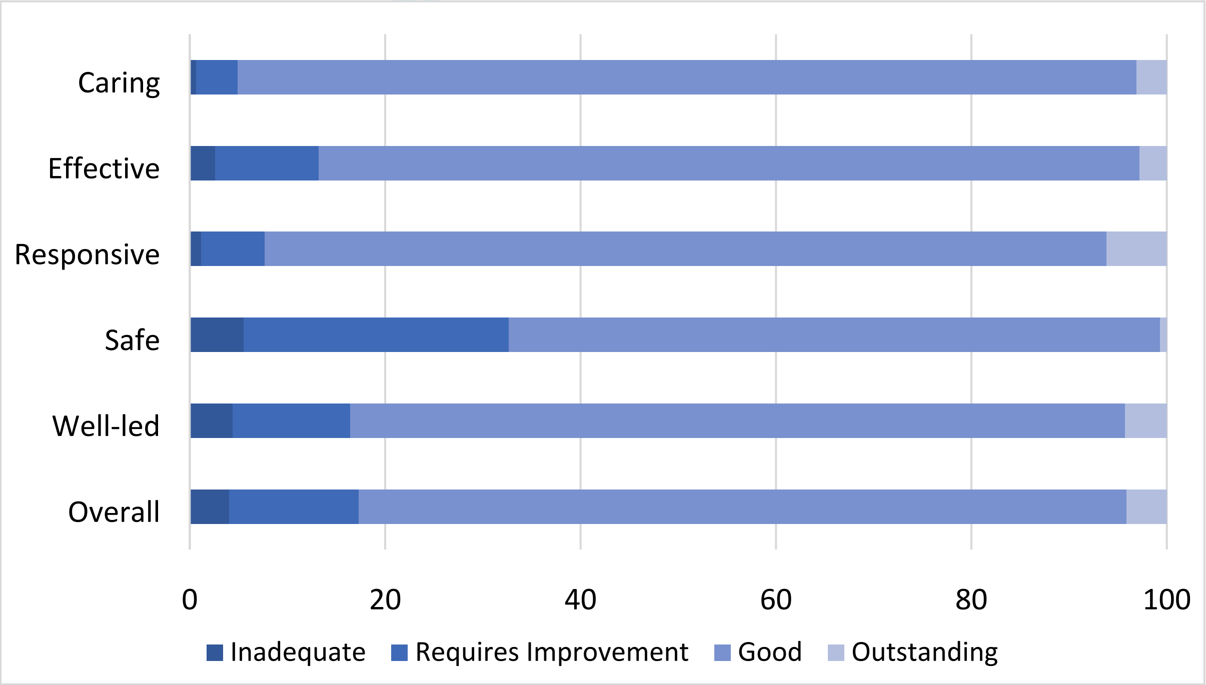

Figure 2: Practice capitation funding by overall practice rating: 'Inadequate' versus 'Outstanding'

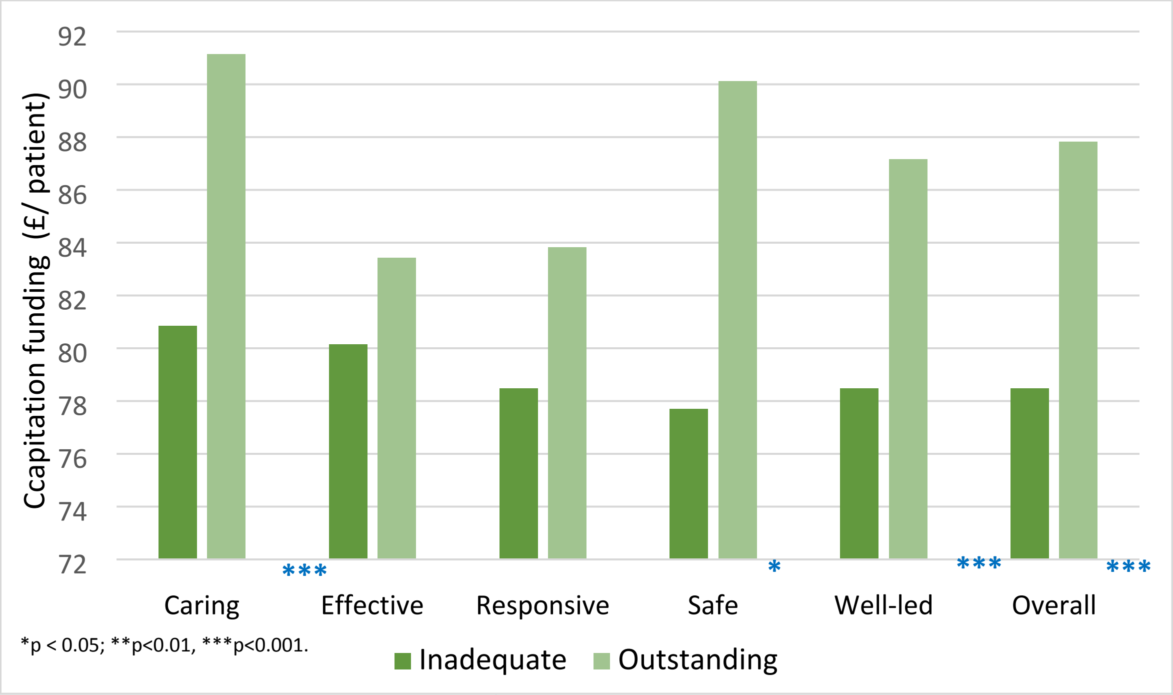

Figure 3: Estimated probability of overall practice rating of 'Inadequate' or 'Outstanding': capitation funding per registered patient

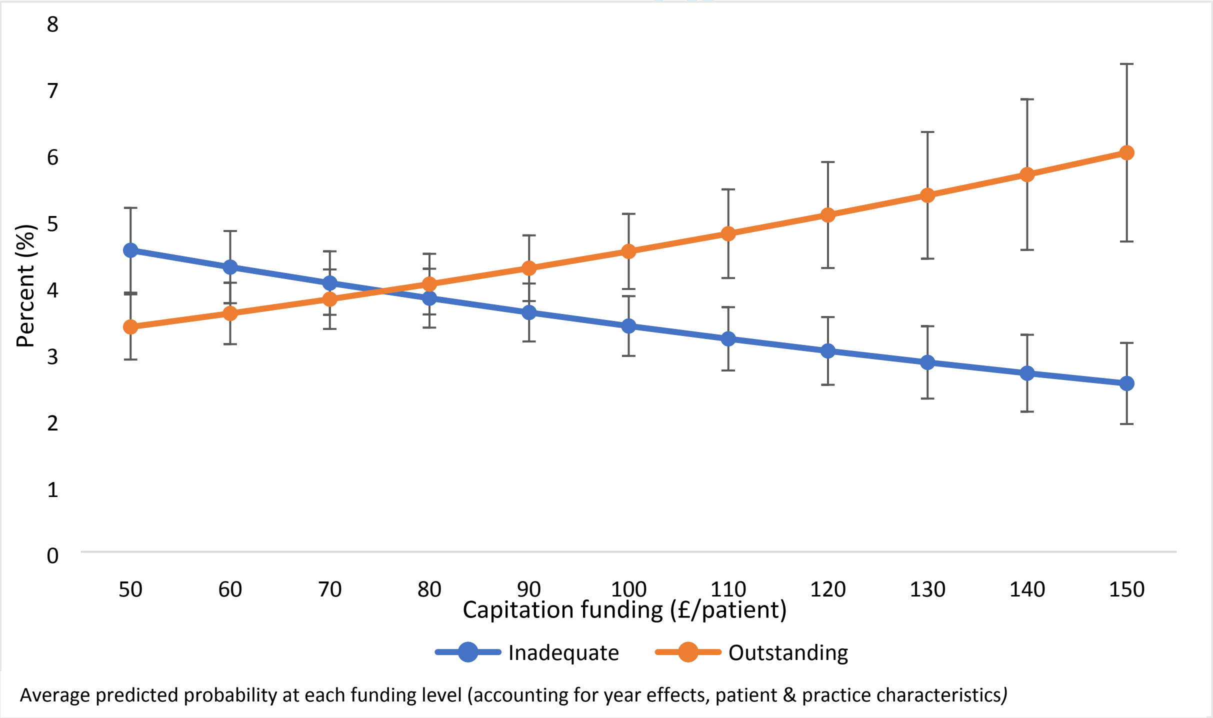

For peer review only - http://bmjopen.bmj.com/site/about/guidelines.xhtml

Figure 4: Estimated probability of overall practice CQC ratings of 'Outstanding': training versus non-training practices

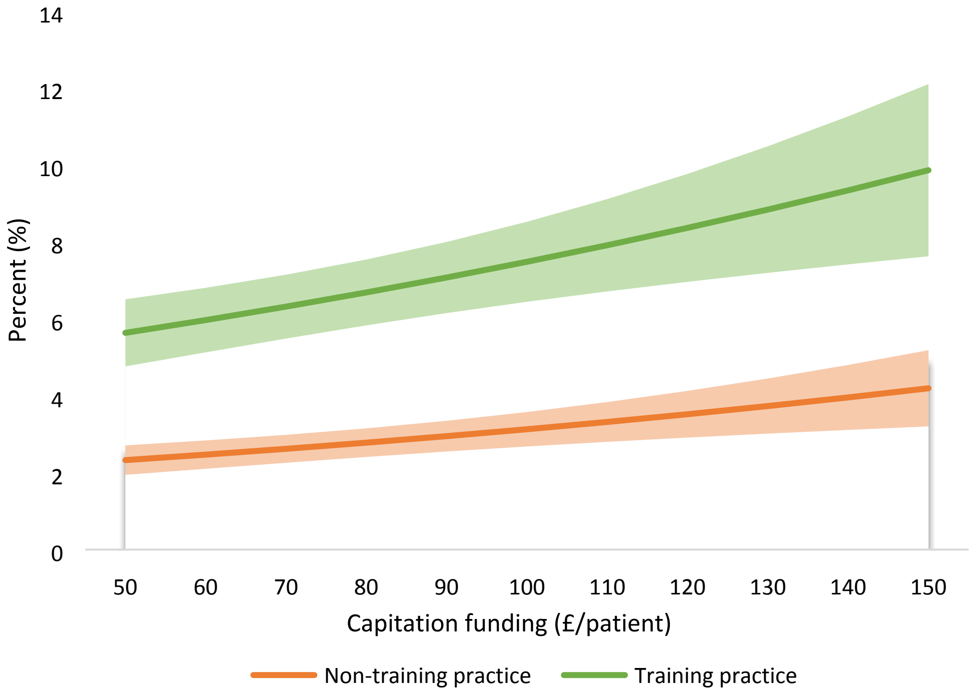

Adjusted for year effects, patient characteristics, practice characteristics & funding
Shaded areas demonstrate 95%CI

Figure 5: Estimated probability of overall practice CQC ratings of 'Outstanding': group and single-handed versus group practices

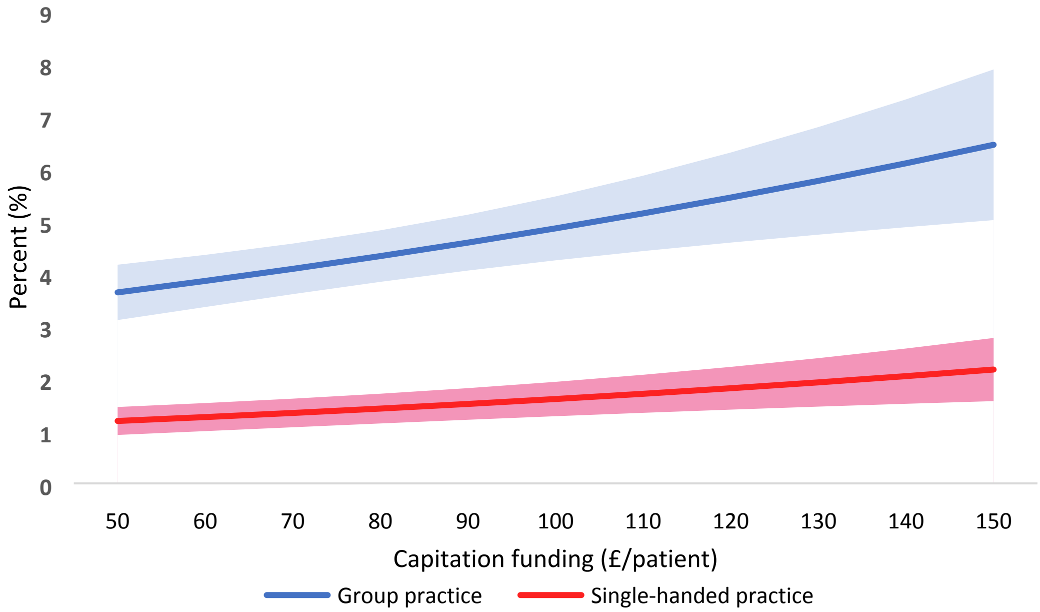

Adjusted for year effects, patient characteristics, practice characteristics & funding
Shaded areas demonstrate 95%CI

Figure 6: Estimated probability of overall practice CQC ratings of 'Outstanding': rural and urban practices

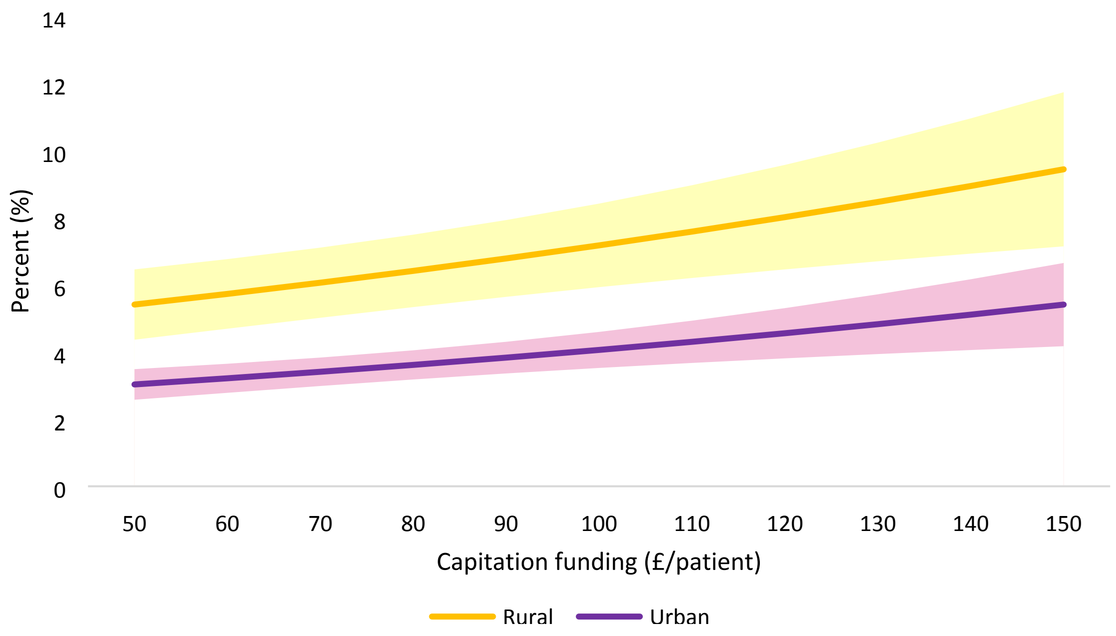

Adjusted for year effects, patient characteristics, practice characteristics & funding
Shaded areas demonstrate 95%CI

For peer review only

Appendix- Table A: Association of general practice funding with overall practice CQC rating: predictor variable in fully adjusted model.

**Characteristics**	**Odds Ratio**	**95% CI**
Capitation funding (per 100 patients)	1.99***	1.34, 2.95
Year 2	1.00	0.98, 1.01
Year 3	0.98	0.96, 1.01
Deprivation	1.00*	1.00, 1.00
Patients aged 0 to 4 years (proportion)	0.79	0.42, 1.47
Patients aged 75 years or old (proportion)	0.80	0.56, 1.15
Patients in nursing home (proportion)	2.27	0.47, 11.01
Patients Asian or black ethnicity (proportion)	0.94*	0.88, 1.00
Region: Midlands§	0.97*	0.95, 0.99
Region: London§	0.95***	0.93, 0.98
Region: South§	0.93***	0.91, 0.96
Minimum distance to hospital	1.00	1.00, 1.00
Rurality	0.96*	0.93, 1.01
Contract type (GMS/PMS)	1.01	0.99, 1.02
Dispensing Practice status (yes/no)	1.01	0.98, 1.03
Singlehanded practice (yes/no)	0.95***	0.92, 0.97
Training Practice status (yes/no)	1.07***	1.05, 1.09
List size per Full Time GP (per 100 patients)	0.98***	0.97, 0.99
List size per Full Time staff (per 100 patients)	0.97*	0.95, 0.99
List size per Full Time Nurse (per 100 patients)	1.00	1.00, 1.00
*p< 0.05; **p< 0.01; ***p< 0.001		

§ Comparator Region: North

For peer review only - http://bmjopen.bmj.com/site/about/guidelines.xhtml

References:

1. Ham C, Berwick D, Dixon J. Improving quality in the English NHS: A strategy for action. London: The Kings Fund, 2016.
2. Department of Health. High Quality Care for All – NHS Next Stage Review Final Report. London: Department of Health, 2008.
3. Campbell S, Steiner A, Robison J, Weber D, Raven A, Roland M. Is the quality of care in general medical practice improving? Results of a longitudinal observational study. Br J Gen Pract 2003;53:298-304.
4. Gillam S, Siriwardena A. Regulation in primary care. Quality in Primary Care 2014;22:57–61.
5. Emmert M, Eijkenaar F, Kemter H, Esslinger AS, Schoffski O. Economic evaluation of pay-for-performance in health care: a systematic review. Eur J Health Econ. 2012;13:755-67.
6. Kontopantelis E., Springate D. A., Ashworth M., Webb R. T., Buchan I. E., Doran T. Investigating the relationship between quality of primary care and premature mortality in England: a spatial whole-population study. BMJ. 2015;350:h904.
7. Campbell SM, Reeves D, Kontopantelis E, Sibbald B, Roland M. Effects of pay for performance on the quality of primary care in England. NEJM. 2009;361:368-78.
8. NHS Digital. NHS Payments to General Practice, England, 2016/17. 2017.
9. British Medical Association. Focus on the global sum allocation formula (Carr-Hill Formula): British Medical Association; 2015 [12th April 2016]. Available from: https://www.google.co.uk/url?sa=t&rct=j&q=&esrc=s&source=web&cd=1&cad=rja&uact=8&ved=0ahUKEwi_sLK47f_OAhWLAMAKHZ2bC8oQFggcMAA&url=https%3A%2F%2Fwww.bma.org.uk%2F-%2Fmedia%2Ffiles%2Fpdfs%2Fpractical%2520advice%2520at%2520work%2Fcontracts%2Fgps%2Ffocus%2520on%2520global%2520sum%2520allocation%2520formula.pdf%3Fla%3Den&usg=AFQjCNEfyEp15cpvpLtT77bIobeHfSmGMQ&sig2=ojUD-oKqav9bcLejniNdtw&bvm=bv.131783435,d.d2s.
10. NHS Digital. General and Personal Medical Services, England: 2005-2015, as at 30 September, Provisional Experimental statistics. NHS Digital, 2016.
11. L'Esperance V, Sutton M, Schofield P, Round T, Malik U, White P, et al. Impact of primary care funding on secondary care utilisation and patient outcomes: a retrospective cross-sectional study of English general practice. Br J Gen Pract. 2017(DOI: https://doi.org/10.3399/bjgp17X693101).
12. Dunham AH, Dunbar JA, Johnson JK, Fuller J, Morgan M, Ford D. What attributions do Australian high-performing general practices make for their success? Applying the clinical microsystems framework: a qualitative study. BMJ Open. 2018;8(4):e020552.
13. Care Quality Commission. The state of care in general practice 2014 to 2017. London: Care Quality Commission, 2017.
14. Care Quality Commission. How CQC monitors, inspects and regulates NHS GP practices. London: Care Quality Commission, 2018.
15. NHS Digital. NHS Payments to General Practice, England: NHS Digital; 2015. Available from: http://digital.nhs.uk/catalogue/PUB18468/nhspaymentsgp-14-15-ann1.xlsx.
16. NHS Digital. General and Personal Medical Services: NHS Digital; 2016. Available from: http://digital.nhs.uk/catalogue/PUB20503.
17. Department for Communities and Local Government. English indices of deprivation 2015. Department for Communities and Local Government 2015.
18. NHS England. The GP Patient Survey. NHS England, 2015 July 2014 Report No.
19. Ashworth M, White P, Jongsma H, Schofield P, Armstrong D. Antibiotic prescribing and patient satisfaction in primary care in England: cross-sectional analysis of national patient survey data and prescribing data. Br J Gen Pract. 2016;66(42).
20. Office for National Statistics. 2011 Census: Office for National Statistics; 2011 [21st January 2016]. Available from: https://www.ons.gov.uk/census/2011census.

For peer review only

21. NHS Digital. NHS Payments to General Practice, England, 2013-14: Experimental Statistics – Report. Available from: 2015 [5th Sept 2015]. Available from: http://www.hscic.gov.uk/catalogue/PUB16847/nhspaymentsgp-13-14-rep.docx. .
22. Ashworth M, Schofield P, Durbaba S, Ahluwalia S. Patient experience and the role of postgraduate GP training: a cross-sectional analysis of national Patient Survey data in England. Br J Gen Pract. 2014;64(620):168-77.
23. Lall R, Campbell MJ, Walters SJ, Morgan K. A review of ordinal regression models applied on health-related quality of life assessments. Stat Methods Med Res. 2002;11(I):49-67.
24. Brant R. Assessing proportionality in the proportional odds model for ordered logistic regression. Biometrics. 1990;46: 1171-8.
25. Roberts MJ, Campbell JL, Abel GA, al et. Understanding high and low patient experience scores in primary care: analysis of patients' survey data for general practices and individual doctors. BMJ. 2014;349:g6034.
26. Ashworth M, Armstrong D. The relationship between general practice characteristics and quality of care: a national survey of quality indicators used in the UK Quality and Outcomes Framework, 2004–5. BMC Fam Pract. 2006;7:68.
27. McLean G, Guthrie B, Mercer SW, Watt GC. General practice funding underpins the persistence of the inverse care law: cross-sectional study in Scotland. Br J Gen Pract. 2015; 65(641):799-805.
28. Levene L, Baker R, Wilson A, Walker N, Boomla K, Bankart M. Population health needs as predictors of variations in NHS practice payments: a cross-sectional study of English general practices in 2013–2014 and 2014–2015. Br J Gen Pract. 2016;67(654):e10-e9.
29. Mukhtar TK, Bankhead C, Stevens S, Perera R, Holt TA, Salisbury C, et al. Factors associated with consultation rates in general practice in England, 2013–2014: a cross-sectional study. Br J Gen Pract 2018;68(670):e370-e7.
30. Lyratzopoulos G, Elliott M, Barbiere JM, al et. Understanding ethnic and other socio-demographic differences in patient experience of primary care: evidence from the English General Practice Patient Survey. BMJ Qual Saf. 2012;21(1):21-9.
31. NHS England. The NHS Long Term Plan. NHS England, 2019.
32. Care Quality Commission. The five key questions we ask: Care Quality Commission; 2016 [12th April 2017]. Available from: http://www.cqc.org.uk/content/five-key-questions-we-ask.

For peer review only

For peer review only - http://bmjopen.bmj.com/site/about/guidelines.xhtml

STROBE Statement—checklist of items that should be included in reports of observational studies

	Item No.	Recommendation	Page No.	Relevant text from manuscript
**Title and abstract**	1	(*a*) Indicate the study's design with a commonly used term in the title or the abstract	1	"Cross-sectional study"
		(*b*) Provide in the abstract an informative and balanced summary of what was done and what was found	1	Summarised in Abstract: Design, Participants, Main outcome measures, Results
**Introduction**				
Background/rationale	2	Explain the scientific background and rationale for the investigation being reported	2-3	The link between measured dimensions of quality and primary care funding data
Objectives	3	State specific objectives, including any prespecified hypotheses	3	"we assess the relationship of practice capitation funding with overall CQC ratings and with the individual CQC domains"
**Methods**				
Study design	4	Present key elements of study design early in the paper	5	Presented under'Sample'.
Setting	5	Describe the setting, locations, and relevant dates, including periods of recruitment, exposure, follow-up, and data collection	3	Presented under 'Data Sources'
Participants	6	(*a*) *Cohort study*—Give the eligibility criteria, and the sources and methods of selection of participants. Describe methods of follow-up<br>*Case-control study*—Give the eligibility criteria, and the sources and methods of case ascertainment and control selection. Give the rationale for the choice of cases and controls<br>*Cross-sectional study*—Give the eligibility criteria, and the sources and methods of selection of participants	5	Cross-sectional study: general practice eligibility criteria summarised under 'Sample'
		(*b*) *Cohort study*—For matched studies, give matching criteria and number of exposed and unexposed<br>*Case-control study*—For matched studies, give matching criteria and the number of controls per case		

For peer review only - http://bmjopen.bmj.com/site/about/guidelines.xhtml

Variables	7	Clearly define all outcomes, exposures, predictors, potential confounders, and effect modifiers. Give diagnostic criteria, if applicable	4/5	Practice variables summarised under the heading 'practice data'; funding variables summarised under the heading 'practice funding data'
Data sources/ measurement	8*	For each variable of interest, give sources of data and details of methods of assessment (measurement). Describe comparability of assessment methods if there is more than one group	3	All sources of data listed under the heading, 'Data Sources'
Bias	9	Describe any efforts to address potential sources of bias	4	The large no. of variables included in our regression model are designed to maximise the explanatory power of the model
Study size	10	Explain how the study size was arrived at	5	Included practices represent ALL practices, minus exclusions based on predetermined exclusion criteria. Only lack of data availability restricted the sample size.

Continued on next page

For peer review only

Quantitative variables	11	Explain how quantitative variables were handled in the analyses. If applicable, describe which groupings were chosen and why	5/6	Quantitative variables analysed according to description in section headed, 'Data Analysis'.
Statistical methods	12	(*a*) Describe all statistical methods, including those used to control for confounding	5/6	Regression modelling – see Data Analysis
		(*b*) Describe any methods used to examine subgroups and interactions	6	Sub-groups such as 'outstanding' or 'inadequate' practices identified
		(*c*) Explain how missing data were addressed	5/6	Practices excluded if missing data
		(*d*) *Cohort study*—If applicable, explain how loss to follow-up was addressed<br>*Case-control study*—If applicable, explain how matching of cases and controls was addressed<br>*Cross-sectional study*—If applicable, describe analytical methods taking account of sampling strategy	n/a	The study was not a sample
		(*e*) Describe any sensitivity analyses	8	See heading 'Sensitivity analyses'
**Results**				
Participants	13*	(a) Report numbers of individuals at each stage of study—eg numbers potentially eligible, examined for eligibility, confirmed eligible, included in the study, completing follow-up, and analysed	5	Summarised under 'sample'. 7310 practices in England; 54 excluded.
		(b) Give reasons for non-participation at each stage	n/a	n/a (exclusion criteria stated above)
		(c) Consider use of a flow diagram	n/a	Exclusion criteria summarised on pg5 without use of a Flow diagram
Descriptive data	14*	(a) Give characteristics of study participants (eg demographic, clinical, social) and information on exposures and potential confounders	13	Table 2 summarises, 'Characteristics of general practices and their populations in England'
		(b) Indicate number of participants with missing data for each variable of interest	n/a	Sample only included those with available data
		(c) *Cohort study*—Summarise follow-up time (eg, average and total amount)	n/a	
Outcome data	15*	*Cohort study*—Report numbers of outcome events or summary measures over time	n/a	
		Case-control study—Report numbers in each exposure category, or summary measures of exposure	n/a	
		Cross-sectional study—Report numbers of outcome events or summary measures	13	Summary measures in Table 2
Main results	16	(*a*) Give unadjusted estimates and, if applicable, confounder-adjusted estimates and their precision (eg, 95% confidence interval). Make clear which confounders were adjusted for and why they were included	14	Table 4 summarises 'unadjusted' and three levels of 'adjusted' analyses

For peer review only - http://bmjopen.bmj.com/site/about/guidelines.xhtml
For peer review only

		15	Confounders adjusted for are listed in Table 5
	(*b*) Report category boundaries when continuous variables were categorized	n/a	No categorisation of continual variables
	(*c*) If relevant, consider translating estimates of relative risk into absolute risk for a meaningful time period	n/a	Absolute financial values are presented in the findings.

Continued on next page

For peer review only

Other analyses	17	Report other analyses done—eg analyses of subgroups and interactions, and sensitivity analyses	8	Sensitivity analyses summarised
**Discussion**				
Key results	18	Summarise key results with reference to study objectives	9	Key finding summarised at opening of Discussion: 'This study has demonstrated that higher capitation funding is associated with significantly higher overall practice quality ratings and ratings across all individual domains.'
Limitations	19	Discuss limitations of the study, taking into account sources of potential bias or imprecision. Discuss both direction and magnitude of any potential bias	10	Discussed under heading, 'Strengths and Weaknesses of the study'
Interpretation	20	Give a cautious overall interpretation of results considering objectives, limitations, multiplicity of analyses, results from similar studies, and other relevant evidence	10/11	Cautious interpretation summarised with reference to the literature
Generalisability	21	Discuss the generalisability (external validity) of the study results	11	Summarised under, 'Implications for policy and practice'
**Other information**				
Funding	22	Give the source of funding and the role of the funders for the present study and, if applicable, for the original study on which the present article is based	In submission details	First author is funded as Doctoral Research Fellow (DRF) by NIHR

*Give information separately for cases and controls in case-control studies and, if applicable, for exposed and unexposed groups in cohort and cross-sectional studies.

Note: An Explanation and Elaboration article discusses each checklist item and gives methodological background and published examples of transparent reporting. The STROBE checklist is best used in conjunction with this article (freely available on the Web sites of PLoS Medicine at http://www.plosmedicine.org/, Annals of Internal Medicine at http://www.annals.org/, and Epidemiology at http://www.epidem.com/). Information on the STROBE Initiative is available at www.strobe-statement.org.

For peer review only

For peer review only - http://bmjopen.bmj.com/site/about/guidelines.xhtml

BMJ Open

The relationship between general practice capitation funding and the quality of primary care in England: a cross-sectional, three-year study

Journal:	*BMJ Open*
Manuscript ID	bmjopen-2019-030624.R1
Article Type:	Original research
Date Submitted by the Author:	13-Aug-2019
Complete List of Authors:	L'Esperance, Veline; King's College London, UK, School of Population Health and Environmental Sciences<br>Gravelle, Hugh; University of York, Centre for Health Economcs<br>Schofield, Peter; King's College London, UK, School of Population Health and Environmental Sciences<br>Santos, Rita; University of York, Centre for Health Economics<br>Ashworth, Mark; King's College London, UK, School of Population Health and Environmental Sciences
**Primary Subject Heading**:	General practice / Family practice
Secondary Subject Heading:	Health economics, Health policy, Health services research
Keywords:	PRIMARY CARE, Quality in health care < HEALTH SERVICES ADMINISTRATION & MANAGEMENT, HEALTH ECONOMICS, Health inequalities, primary care funding, general practice

SCHOLARONE™
Manuscripts

Title:

The relationship between general practice capitation funding and the quality of primary care in England: a cross-sectional, three-year study

Authors:

Veline L'Esperance[1], Hugh Gravelle[2], Peter Schofield[3], Rita Santos[4], Mark Ashworth[5]

1. Doctoral Research Fellow, School of Population Health & Environmental Sciences, Kings College London, London, UK
2. Professor of Economics, Centre for Health Economics, University of York, York, UK
3. Wolfson Lecturer, School of Population Health & Environmental Sciences, Kings College London, London, UK
4. Research Fellow, Centre for Health Economics, University of York, York, UK
5. Reader in Primary Care, School of Population Health & Environmental Sciences, Kings College London, London, UK

Corresponding author email: mark.ashworth@kcl.ac.uk

Abstract:

Objective To explore the relationship between general practice capitation funding and quality ratings based on general practice inspections.

Design Cross-sectional study pooling three years of primary care administrative data.

Setting UK primary care.

Participants 7310 practices (95% of all practices) in England which underwent Care Quality Commission (CQC) inspections between November 2014 and December 2017.

Main outcome measures CQC ratings. Ordered logistic regression methods were used to predict the relationship between practice capitation funding and CQC ratings in each of five domains of quality: caring, effective, responsive, safe and well-led, together with an overall practice rating.

Results Higher capitation funding per patient was significantly associated with higher CQC ratings across all five quality domains; caring (odds ratio [OR] 1.14, 95% confidence interval [CI]: 1.04 to 1.23); effective (OR 1.08, 95% CI: 1.00 to 1.16); responsive (OR 1.09, 95%CI:

For peer review only - http://bmjopen.bmj.com/site/about/guidelines.xhtml

1.02 to 1.17); safe (OR 1.11, 95% CI: 1.05 to 1.18); well-led (OR 1.13, 95% CI: 1.06 to 1.20); and overall rating (OR 1.13, 95% CI: 1.06 to 1.19).

Conclusion: Higher capitation funding was consistently associated with higher ratings across all CQC domains and in the overall practice rating. This study suggests that measured dimensions of the quality of care are related to the underlying capitation funding allocated to each general practice, implying that additional capitation funding may be associated with higher levels of primary care quality.

Strengths and limitations

- A cross-sectional study covering three years of primary care data
- The definition of primary care quality used in this study was multidimensional, based on inspection findings and covering patient safety, patient experience, clinical effectiveness.
- The association between the achievement of quality ratings and practice capitation funding was explored, adjusted for known confounders
- Although based on a near complete sample of general practices in England, bias may have been introduced by data coding and recording errors
- Longer term and prospective studies are required to strengthen causal inferences

For peer review only - http://bmjopen.bmj.com/site/about/guidelines.xhtml

Introduction

Improving the quality of care is a major focus of UK government health policy(1). High quality health care has three main components: clinical achievement, patient experience and patient safety(2). There is wide variation between general practices in the achievement of clinical care quality indicators and patient reported satisfaction(3, 4).

It is important to understand whether variations in the quality of care provided across practices are related to variations in their funding. Healthcare quality regulation in England is currently undertaken by the Care Quality Commission, focuses on outcomes for patients and has a wide range of enforcement powers, including closure and deregistration of services, if essential standards are not met.(5)

Studies of the relationship between quality and funding in English general practices have largely focussed on the Quality and Outcomes Framework (QOF) which rewards practices for higher quality care, as defined by the achievement of clinical process and outcome targets. The QOF has had limited impact on reducing secondary care costs(6) or improving primary care performance(7, 8). In terms of financial incentivisation, the QOF accounted for approximately 7.8% of funding received by general practices in England in 2016(9). In contrast, capitation payments represent the largest proportion of funding to general practice (54% in 2016) and are related to the number of registered patients in each practice(9), adjusted for factors thought to increase the demand on primary care services(10). Other components of general practice funding include additional payments for postgraduate training, the provision of additional clinical services ('enhanced services') and various

For peer review only - http://bmjopen.bmj.com/site/about/guidelines.xhtml

reimbursements to cover the costs of premises, computers and for some practices, dispensing medication(11).

Greater capitation spending on general practices has been found to be associated with reductions in secondary care usage and costs, and increased patient satisfaction(12). Studies have also shown that leadership within the practice organisation plays a key role in the delivery of high quality care(13). Until recently, nationally derived metrics of inspection-based primary care quality were unavailable. Since October 2014 all general practices have been subjected to inspections by the Care Quality Commission (CQC) (14), (5). The CQC reports on the extent to which practices are caring, effective, responsive to the needs of patients, safe, and well-led(5, 15) and also combines these five domains to produce an overall practice rating. These five domains incorporate components of clinical achievement, patient experience and patient safety(2). In this study, we assess the relationship of practice capitation funding with overall CQC ratings and with the individual CQC domains. We aimed to examine the relationship between practice funding and the quality of care as determined by inspection-based quality assessment. Analysis of total practice funding would have introduced confounding through inclusion of quality-related payments. We therefore used capitation funding as our measure of practice funding since this financial indicator is independent of financial rewards associated with quality achievement such as the QOF and other national and local incentive schemes.

Methods

Data sources

We linked practice-level data on NHS payments to general practice identifiers(16), CQC inspection ratings(15), NHS administrative datasets, General and Personal Medical Services

For peer review only - http://bmjopen.bmj.com/site/about/guidelines.xhtml

Statistics (17), and small area Census and socio-economic data from Neighbourhood Statistics(18).

Care Quality Commission Ratings

CQC ratings are based on publicly available data (such as QOF and General Practice Patient Survey(19)), practice inspections, interviews with patients and staff, complaints, clinical record reviews, reviews of practice documents and policies(15). We used CQC ratings for practices with completed CQC reports first inspected between November 2014 and December 2017 (n = 7310, 95% of all practices). Practice ratings were obtained from the CQC; these data are publicly available on request. For practices which required reinspection only the first inspection score was included in the analysis. The five domains of quality described by CQC inspections are summarised in Table 1; each is rated on a 4-point scale.

Practice data

Data for all general practices in England were obtained from the General and Personal Medical Services database, for 2014/15, 2015/16 and 2016/17 financial years (17). These data are publicly available from NHS Digital. Our use of practice based demographic data followed a previously used methodology(20). Patient characteristics included the proportion of patients aged 0 to 4 years, proportion of patients aged 75 years or older and proportion of nursing home patients. Deprivation data for each general practice was attributed as the mean of the Index of Multiple Deprivation (IMD) 2015(18) weighted by the proportion of practice patients resident in each Lower Layer Super Output area (LLSOA). Neighbourhood ethnicity (proportion Asian or black) derived from the 2011 national census, was attributed to practices weighted by the proportion of the practice population in each LLSOA(21). The following

For peer review only - http://bmjopen.bmj.com/site/about/guidelines.xhtml

practice characteristics were included: region (North, Midlands, London and South), contract type (General Medical Services or Personal Medical Services), minimum distance from an acute hospital, dispensing status (whether the practice dispensed as well as prescribed medication), singlehanded practice status (singlehanded practices have ≤1.0 FTE GP; group practices have >1.0 FTE GPs) and training practice status. We did not include practice staffing (GPs, nurses, other staff) as explanatory variables in the model because staffing is likely to be directly affected by practice capitation funding and so inclusion of these variable may lead to an underestimate of the full effect of capitation funding. Moreover, a major change in the way in which staffing data was collected in 2015/16 would have resulted in a large reduction in observation number.

Practice capitation

Practice capitation funding depends on the total number of practice patients adjusted to reflect factors affecting GP workload (age, gender, patients in nursing and residential homes, small area measures of morbidity), rurality and an index of local staff costs which affect the cost of providing services(10). Data was available for the financial years 2014/15, 2015/16 and 2016/7(22). We use the mean capitation payment per patient for the year prior to inspection and the year in which the practice was inspected.

Sample

We linked inspected practices (n=7310) with funding data for their year of inspection. We excluded atypical practices with ≤750 registered patients (n=10) or ≤500 patients per FTE GP (n= 8) following a previously used method(23). Practices with recorded negative (n= 2) or zero funding (n = 52) were excluded. The final analysis sample consisted of 7238 practices.

For peer review only - http://bmjopen.bmj.com/site/about/guidelines.xhtml

Data Analysis

Analysis was conducted at GP practice level. Since the CQC rating outcomes are ordered categories we used ordered logistic regression to model the relationship between funding and the practice CQC ratings(24). Separate models were estimated for each domain.

The key explanatory variable was capitation funding per patient. Capitation funding per patient is reported in standard deviation units. In addition to patient and practice characteristic covariates, the regression models included year effects to allow for inspection year and annual general practice funding uplifts. We accounted for local area effects by adjusting for clustering at Clinical Commissioning Group (CCG) level. Multicollinearity was tested for by calculating the Variance Inflation Factor (VIF) and variables with a value for VIF>5 were excluded. The proportional odds assumption of the ordered logit model was also tested(25). We report the odds ratio from the estimated models.

We calculated the average marginal effects of funding on the predicted probabilities of achieving overall ratings of “outstanding” and “inadequate” for all practices. We also compared the predicted probabilities of an “outstanding” overall rating at different practice capitation funding levels for training versus non-training practices, single-handed versus multi-handed practices, and rural versus urban practices. STATA 14 (StataCorp, College Station, TX) was used for all statistical analyses.

Patient involvement

For peer review only - http://bmjopen.bmj.com/site/about/guidelines.xhtml

Funding for this study included funding of a dedicated patient involvement group. Patients were involved in developing plans for the study design, approving the outcome measures and commenting on the potential impact of outcomes. A lay summary was also provided.

Results

Summary statistics for the main characteristics of the general practices are provided in Table 2. Mean practice capitation funding per registered patient increased from £77.49 in 2014/5 to £83.17 in 2016/7 (Table 3). The mean capitation funding per patient across the CQC inspection period was £79.48. The standard deviation of the mean capitation funding per patient was £22.00.

The distribution of practice ratings across each quality domain is shown in Figure 1. A total of 79% (n = 5774) of practices achieved an overall rating of 'Good', while only 4% (294) achieved an overall rating of 'Outstanding'. 'Inadequate' ratings varied across the domains, from 1% (caring domain) to 6% (safety domain) and 4% (overall).

Figure 2 shows the difference in capitation funding for practices with the lowest quality rating compared to those with the highest quality rating. In each domain, 'Inadequate' practices received less capitation funding. Using an independent group t-test this difference was found to be significant for three domains (caring, safe, well-led) and for the overall practice rating.

Table 4 reports the odds ratios on capitation funding per patient estimated from four regression models of overall practice CQC rating. The odds ratio on capitation funding per patient is reported in standard deviation (SD units). In the first model capitation funding is the only explanatory variable (unadjusted model); remaining models are adjusted for

For peer review only - http://bmjopen.bmj.com/site/about/guidelines.xhtml

inclusion of successive additional explanatory variables: year effects, patient characteristics, and practice characteristics. The unadjusted model shows an association between higher capitation funding and higher overall CQC ratings with an odds ratio (OR) of 1.09 (95% confidence interval (CI): 1.03 to 1.15). Allowing for the year of inspection increased the OR slightly to 1.10 (95%CI: 1.04 to 1.16). Additional allowance for patient characteristics (OR 1.13; 95%CI: 1.06 to 1.19) and practice characteristics (OR 1.13; 95%CI: 1.06 to 1.19) further increased the odds ratio. The number of observations in Table 4 fell from 7,168 to 7,045 because of missing data. Very similar changes in odds ratios across the models were observed when all models were restricted to equal sample sizes. A likelihood ratio test demonstrated that the addition of patient and practice variables create a statistically significant improvement in model fit, confirming that higher odds ratios were associated with the addition of model variables, rather than to a change in sample size.

The final adjusted model indicates that for a one standard deviation increase in capitation funding, the odds of achieving an outstanding CQC rating are 1.13 times greater, given that other variables are held constant. We have also shown the estimated changes in the probabilities of achieving ‘inadequate’ and ‘outstanding’ CQC ratings implied by this model in Figures 3 to 7.

Table 5 reports odds ratios for all the explanatory variables in the overall practice quality rating model (Model 4, Table 4). In addition to higher practice capitation funding, rural practice and training practice status were significantly associated with higher overall practice ratings. For example, the adjusted odds ratio of a training practice achieving an ‘outstanding’

For peer review only - http://bmjopen.bmj.com/site/about/guidelines.xhtml

CQC rating is 2.30 times greater than for a non-training practice. Conversely, for singlehanded practices the odds of achieving an ‘outstanding’ rating is 0.53 times that for group practices.

The odds ratios for capitation funding per patient from the full models for each CQC domain are shown in Table 6. Higher capitation funding was significantly associated with higher CQC ratings across all five quality domains.

We used the results from the ordered logistic regression models with the full set of explanatory variables to calculate the probability of achieving an overall practice rating of ‘Outstanding’ or ‘Inadequate’ at different levels of capitation funding. Figure 3 shows the average predicted probability of achieving an ‘Outstanding’ rating for a range of per capita funding levels. The probabilities are the average of the estimated probabilities for each practice calculated at each funding level using actual values of the practice non-funding characteristics (year effects, patient characteristics and practice characteristics). Figure 4 shows the average predicted probability of achieving an ‘Inadequate’ practice rating. Higher capitation funding was associated with reduced probability of achieving an ‘Inadequate’ rating and increased probability of an ‘Outstanding’ quality rating. At capitation payments above £100 per patient, practices have a greater probability of being rated as “Outstanding” rather than “Inadequate”.

We also compared the probability of achieving an ‘Outstanding’ rating at different levels of practice capitation funding for training versus non-training practices (Figure 5), for single-handed versus group practices (Figure 6), and for rural versus urban practices (Figure 7). At all levels of funding, the probability of achieving an ‘Outstanding’ rating is higher for training practices than non-training practices, for group practices than single handed practices, and

For peer review only - http://bmjopen.bmj.com/site/about/guidelines.xhtml

rural practices than urban practices. In all cases higher capitation funding is associated with higher probabilities of an 'Outstanding' rating.

Sensitivity Analyses

The Brant test(25) assesses the proportional odds assumption that the distance between each category is equivalent. Four of the variables included in our model (region, proportion of patients aged 0-4 years, contract type and single-hander status) did not meet the assumption of proportionality of the odds ratios. However, our variable of interest, capitation funding per patient, did not violate the proportional odds assumption. A partial proportional odds model excluding these four variables, estimated by generalised ordered logistic regression, yielded similar results to our main model: higher capitation funding was significantly associated with increase probability of achieving an 'Outstanding' rating (OR 1.14, 95% CI: 1.04 to 1.25).

Discussion

This study has demonstrated that higher capitation funding is associated with significantly higher overall practice quality ratings and ratings across all individual domains.

Practice characteristics such as post-graduate training practice and group practice status were also associated with higher quality ratings, representing primary care structures which support higher quality of care. However, some factors related to the registered practice population, such as urban location, social deprivation and larger proportions of ethnic minority patients were negatively associated with the practice quality of care rating. Many of these factors are already known to be negatively associated with reported patient satisfaction (26) and QOF achievement (27). Including them in the model led to a stronger association of practice capitation funding with practice quality rating. The likely reason for this is that

For peer review only - http://bmjopen.bmj.com/site/about/guidelines.xhtml

practice capitation funding is positively correlated with patient characteristics which have negative effects on the quality rating. Thus, including these patient characteristics in the model removes a source of bias from omitted variables which would otherwise tend to underestimate the positive association of funding with the quality rating.

Strengths and weaknesses of the study

This is the first study to explore the relationship between practice-level capitation funding and practice quality as measured by CQC ratings. The findings are based on a near complete sample of general practices across England. Using data linkages from a wide range of sources, and multilevel statistical models, this study has been able to demonstrate the independent effects of practice funding and practice characteristics on quality ratings, which might otherwise be confounded in single-level analyses. A variety of sensitivity analyses have confirmed the robustness of the ordered logistic regression modelling.

However, there are several limitations. Routinely collected data are subject to coding and recording errors. As with all observational studies, significant associations, even if large, may not be causal. Although a wide range of potential confounders were included in the models, confounding by omitted variables cannot be excluded.

Comparison with existing literature

These findings complement those of a previous study which found that increased general practice capitation funding was associated with reduced emergency hospital admissions and Accident and Emergency attendances(12).

For peer review only - http://bmjopen.bmj.com/site/about/guidelines.xhtml

In a country level European analysis it was found that systems relying on capitation funding were more responsive than those based on fee for service or mixed payment(28). However, analysis of Scottish general practices suggests that capitation funding may contribute to the persistence of the inverse care law with deprived areas experiencing lower quality of care, as defined by inspection ratings(29). Consistent with our study, others have found that GP practice funding is negatively correlated with healthcare need predictors such as deprivation and non-white ethnicity(30). Previous studies have also demonstrated that greater GP workload may be associated with higher levels of social deprivation and with a higher proportion of Asian patients(31). Similarly, practices with a greater proportion of ethnically diverse patients reported worse patient experience(32). Our work is also consistent with a recent study which demonstrated that GPs co-located with other GPs and professionals had improved outcomes compared with single-handed GP practices such as broader provision of technical procedures, wider coordination with secondary care and increased collaboration among different providers (33).

Our study was based on funding data for general practices but was unable to study the relationship between quality ratings and individual GP income. However, values for overall 'profit' per practice are expected to become available in due course. Other studies have confirmed that incentives based on personal income may influence both quality achievement and productivity(34).

Implications for policy and practice

This work provides further evidence of the association between general practice capitation funding and the quality of primary care. A causal association is plausible and supports the

For peer review only - http://bmjopen.bmj.com/site/about/guidelines.xhtml

argument that increased quality and safety of patient care may be achieved through additional investment. The recently published NHS Long Term Plan(35) outlines proposals to offer increases in capitation payment together with an emphasis on inter-practice cooperation through the formation of primary care networks. Both factors are likely to influence the relationship between funding and the quality of primary care and will require further study. Our findings suggest that revisions to the primary care capitation formula are necessary to ensure that additional funding is provided in urban areas of high deprivation and ethnic minority populations in order to address quality of care inequalities.

Unanswered questions and future research

Future research could extend similar analyses to subsequent 3-year cycles of quality inspection. A longitudinal approach, relating changes in funding to changes in outcomes, is likely to provide more accurate estimates of the effect of funding. Complementary qualitative analysis is likely to provide insight into mechanisms underlying the link between better funded practices and higher quality rating achievement.

Conclusion

Higher capitation funding was consistently associated with higher overall and domain quality ratings yielded by CQC inspections. This study suggests that measured and inspected dimensions of the quality of care are related to the underlying funding allocated to each general practice, implying that additional funding may be associated with higher levels of primary care quality.

What is already known on this topic

- Few studies on the relationship between capitation funding and quality

For peer review only - http://bmjopen.bmj.com/site/about/guidelines.xhtml

- In England, the QOF has had limited impact on reducing healthcare costs and improving primary care performance

What this study adds

- Higher capitation funding is associated with increased quality ratings across all domains
- Since the NHS Long Term Plan calls for increased funding to primary care it is important to understand how and where this funding should be directed
- Our findings imply that further primary care funding is particularly needed in areas of high deprivation and ethnic minority populations where funding may be insufficient to support high quality of care.

For peer review only

For peer review only - http://bmjopen.bmj.com/site/about/guidelines.xhtml

Contributors: VL, HG, PS, RS and MA contributed to the idea and design of the study. VL and PS led on data analysis with statistical advice from HG, RS and MA. VL produced the first draft of the paper; all co-authors contributed and approved the final draft. VL is the guarantor. The corresponding author attests that all listed authors meet authorship criteria and that no others meeting the criteria have been omitted.

Funding: This work was supported by the National Institute for Health Research (NIHR) who funded a Doctoral Research Fellowship for VL (reference, DRF-2017-10-132) and for RS (reference, DRF-2014-07-055). HG and RS were funded by the UK NIHR Policy Research Programme (Policy Research Unit in the Economics of Health and Social Care Systems: Ref 103/0001). The views expressed are those of the authors and not necessarily those of the NHS, the National Institute for Health Research, the Department of Health and Social Care or its arm's length bodies or other UK government departments. The findings presented are independent from the funders who have had no role in study design, data collection, data analysis, data interpretation, or writing of the report.

Competing interests: All authors have completed the ICMJE uniform disclosure form at www.icmje.org/coi_disclosure.pdf and declare: no support from any organisation for the submitted work; no financial relationships with any organisations that might have an interest in the submitted work in the previous three years; no other relationships or activities that could appear to have influenced the submitted work.

Ethical approval: Ethical approval not required for the use of aggregate practice level data as included in this study.

Dissemination declaration: the results have been disseminated to the patient involvement group involved in study design and commenting on potential impact of outcomes.

Data sharing: No additional data available.

For peer review only - http://bmjopen.bmj.com/site/about/guidelines.xhtml

Figures and Tables

Table 1: The five key domains for CQC Inspections

Domain	Description
**Safe**	Patients are protected from abuse and avoidable harm
**Effective**	Care, treatment and support achieves good outcomes, helps patients to maintain quality of life and is based on the best available evidence
**Caring**	Staff involve and treat patients with compassion, kindness, dignity and respect
**Responsive**	Services are organised so that they meet patients' needs
**Well-led**	The leadership, management and governance of the organisation make sure it's providing high-quality care that's based around the individual needs, that it encourages learning and innovation, and that it promotes an open and fair culture

Adapted from: CQC. The five key questions we ask(36)

Table 2. Characteristics of general practices and their populations in England

Variable	Mean	(5th, 95th centiles)
Patient adjusted Index of Multiple Deprivation, 2015	24.5	8.2, 46.1
Proportion of patients aged 0 to 4 years (%)	5.9	3.7, 8.8
Proportion of patients aged 75 years or older (%)	7.7	2.6, 12.9
Proportion of patients: nursing home residents (%)	0.5	0, 1.4
Proportion of patients: Asian or black ethnicity (%)	13.1	0.1, 53.1
List size per full-time equivalent (FTE) GP	1950	1066, 3315
List size per FTE non-clinical employed staff	703	392, 1103
List size per FTE nurse	7166	2810, 15507
Minimum distance of practice from acute hospital (km)	3.8	0.4, 11.8
Proportion of practices by rurality (%)		
Urban	85.5	
Rural: hamlet, village, town & fringe	14.5	
Proportion of practices by region (%)		
North	30.3	
Midlands	29.4	
London	18.0	
South	22.3	
Proportion of practices by contract type (%)		
General Medical Services	59.4	
Personal Medical Services	40.6	
Proportion of dispensing practices (%)	14.6	
Proportion of singlehanded practices (%)	13.1	
Proportion of training practices (%)	30.4	

For peer review only - http://bmjopen.bmj.com/site/about/guidelines.xhtml

For peer review only

Table 3: Capitation funding per registered patient for inspected practices

Inspection year	N	Mean capitation funding	(5th, 95th centiles)
2014/15	2232	£77.49	£59.54, £99.99
2015/16	3790	£80.86	£66.57, £101.66
2016/17	1148	£83.71	£67.74, £106.76

Table 4: Association of capitation funding per patient with overall practice CQC rating: unadjusted and adjusted regression models

	Model 1	Model 2	Model 3	Model 4
Capitation funding odds ratio[1]	1.09**	1.10**	1.13***	1.13***
95% CI of odds ratio	1.03, 1.15	1.04, 1.16	1.06, 1.19	1.06, 1.19
Observations	7,168	7,168	7,144	7,045
Models contain				
Year effects	N	Y	Y	Y
Patient characteristics[2]	N	N	Y	Y
Practice characteristics[3]	N	N	N	Y

1. Odds ratios based on Standard Deviation units.
2. Patient adjusted deprivation, proportion of patients aged 0 to 4 years, proportion of patients aged ≥75 years, proportion patients black or Asian ethnicity, proportion of nursing home residents.
3. Region, minimum distance to hospital, contract type, dispensing status, training practice status, singlehanded

*p< 0.05; **p< 0.01; ***p< 0.001

For peer review only

Table 5: Association of capitation funding with overall practice CQC rating: predictor variable in fully adjusted model.

Characteristics	Odds Ratio	95% CI
Capitation funding per patient (SD units)[1]	1.13***	1.06, 1.19
Year 2	0.92	0.80, 1.05
Year 3	0.76**	0.64, 0.91
Deprivation	0.99**	0.98, 0.99
Patients aged 0 to 4 years (proportion)	1.00	0.95, 1.05
Patients aged 75 years or old (proportion)	0.99	0.96, 1.17
Patients in nursing home (proportion)	1.13*	1.02, 1.26
Patients Asian or black ethnicity (proportion)	0.99*	0.99, 1.00
Region: Midlands[§]	0.64***	0.55, 0.76
Region: London[§]	0.56***	0.93, 0.98
Region: South[§]	0.48**	0.40, 0.58
Minimum distance to hospital	1.00	1.00, 1.00
Rurality (Yes/No)	1.50**	1.18, 1.92
Contract type (GMS/PMS)	1.08	0.96, 1.23
Dispensing Practice status (yes/no)	1.10	0.88, 1.38
Singlehanded practice (yes/no)	0.53***	0.44, 0.63
Training Practice status (yes/no)	2.30***	1.99, 2.65
*p< 0.05; **p< 0.01; ***p< 0.001		

[1] Odds ratios based on Standard Deviation units.
[§] Comparator Region: North

For peer review only - http://bmjopen.bmj.com/site/about/guidelines.xhtml

Table 6: Ordered logistic models: effect of capitation funding on each CQC domain rating

Domains	Odds Ratio[1]	95% CI
Caring	1.14**	1.04, 1.23
Effective	1.08*	1.00, 1.16
Responsive	1.09*	1.02, 1.17
Safe	1.11*	1.05, 1.18
Well-led	1.13***	1.06, 1.20
Overall	1.13***	1.06, 1.19

Adjusted for year effects, patient characteristics & practice characteristics &
[1] Odds ratios based on Standard Deviation units.
*p< 0.05; **p< 0.01; ***p< 0.001

Figure 1: Distribution of CQC ratings by each domain

Figure 2: Practice capitation funding by overall practice rating: 'Inadequate' versus 'Outstanding

Figure 3: Estimated probability of overall practice rating of 'Outstanding' at various levels of capitation funding per registered patient

Figure 4: Estimated probability of overall practice rating of 'Inadequate' at various levels of capitation funding per registered patient

Figure 5: Estimated probability of 'Outstanding' overall practice CQC rating: training versus non-training practices

Figure 6: Estimated probability of 'Outstanding' overall practice CQC rating: single-handed versus group practices

Figure 7: Estimated probability of 'Outstanding' overall practice CQC rating: rural and urban practices

For peer review only - http://bmjopen.bmj.com/site/about/guidelines.xhtml

For peer review only

References:

1. Ham C, Berwick D, Dixon J. Improving quality in the English NHS: A strategy for action. London: The Kings Fund, 2016.
2. Department of Health. High Quality Care for All – NHS Next Stage Review Final Report. London: Department of Health, 2008.
3. Campbell S, Steiner A, Robison J, Weber D, Raven A, Roland M. Is the quality of care in general medical practice improving? Results of a longitudinal observational study. Br J Gen Pract 2003;53:298-304.
4. Llanwarne NR, Abel GA, Elliott MN, Paddison CAM, Lyratzopoulos G, Campbell JL, et al. Relationship Between Clinical Quality and Patient Experience: Analysis of Data From the English Quality and Outcomes Framework and the National GP Patient Survey. Annals of Family Medicine. 2013;11(5):467-72.
5. Gillam S, Siriwardena A. Regulation in primary care. Quality in Primary Care 2014;22:57–61.
6. Emmert M, Eijkenaar F, Kemter H, Esslinger AS, Schoffski O. Economic evaluation of pay-for-performance in health care: a systematic review. Eur J Health Econ. 2012;13:755-67.
7. Kontopantelis E., Springate D. A., Ashworth M., Webb R. T., Buchan I. E., Doran T. Investigating the relationship between quality of primary care and premature mortality in England: a spatial whole-population study. BMJ. 2015;350:h904.
8. Campbell SM, Reeves D, Kontopantelis E, Sibbald B, Roland M. Effects of pay for performance on the quality of primary care in England. NEJM. 2009;361:368-78.
9. NHS Digital. NHS Payments to General Practice, England, 2016/17. 2017.
10. British Medical Association. Focus on the global sum allocation formula (Carr-Hill Formula): British Medical Association; 2015 [12th April 2016]. Available from: https://www.google.co.uk/url?sa=t&rct=j&q=&esrc=s&source=web&cd=1&cad=rja&uact=8&ved=0ahUKEwi_sLK47f_OAhWLAMAKHZ2bC8oQFggcMAA&url=https%3A%2F%2Fwww.bma.org.uk%2F-%2Fmedia%2Ffiles%2Fpdfs%2Fpractical%2520advice%2520at%2520work%2Fcontracts%2Fgps%2Ffocus%2520on%2520global%2520sum%2520allocation%2520formula.pdf%3Fla%3Den&usg=AFQjCNEfyEp15cpvpLtT77bIobeHfSmGMQ&sig2=ojUD-oKqav9bcLejniNdtw&bvm=bv.131783435,d.d2s.
11. NHS Digital. General and Personal Medical Services, England: 2005-2015, as at 30 September, Provisional Experimental statistics. NHS Digital, 2016.
12. L'Esperance V, Sutton M, Schofield P, Round T, Malik U, White P, et al. Impact of primary care funding on secondary care utilisation and patient outcomes: a retrospective cross-sectional study of English general practice. Br J Gen Pract. 2017(DOI: https://doi.org/10.3399/bjgp17X693101).
13. Dunham AH, Dunbar JA, Johnson JK, Fuller J, Morgan M, Ford D. What attributions do Australian high-performing general practices make for their success? Applying the clinical microsystems framework: a qualitative study. BMJ Open. 2018;8(4):e020552.
14. Care Quality Commission. The state of care in general practice 2014 to 2017. London: Care Quality Commission, 2017.
15. Care Quality Commission. How CQC monitors, inspects and regulates NHS GP practices. London: Care Quality Commission, 2018.
16. NHS Digital. NHS Payments to General Practice, England: NHS Digital; 2015. Available from: http://digital.nhs.uk/catalogue/PUB18468/nhspaymentsgp-14-15-ann1.xlsx.
17. NHS Digital. General and Personal Medical Services: NHS Digital; 2016. Available from: http://digital.nhs.uk/catalogue/PUB20503.
18. Department for Communities and Local Government. English indices of deprivation 2015. Department for Communities and Local Government 2015.
19. NHS England. The GP Patient Survey. NHS England, 2015 July 2014 Report No.
20. Ashworth M, White P, Jongsma H, Schofield P, Armstrong D. Antibiotic prescribing and patient satisfaction in primary care in England: cross-sectional analysis of national patient survey data and prescribing data. Br J Gen Pract. 2016;66(42).

For peer review only - http://bmjopen.bmj.com/site/about/guidelines.xhtml

21. Office for National Statistics. 2011 Census: Office for National Statistics; 2011 [21st January 2016]. Available from: https://www.ons.gov.uk/census/2011census.
22. NHS Digital. NHS Payments to General Practice, England, 2013-14: Experimental Statistics – Report. Available from: 2015 [5th Sept 2015]. Available from: http://www.hscic.gov.uk/catalogue/PUB16847/nhspaymentsgp-13-14-rep.docx. .
23. Ashworth M, Schofield P, Durbaba S, Ahluwalia S. Patient experience and the role of postgraduate GP training: a cross-sectional analysis of national Patient Survey data in England. Br J Gen Pract. 2014;64(620):168-77.
24. Lall R, Campbell MJ, Walters SJ, Morgan K. A review of ordinal regression models applied on health-related quality of life assessments. Stat Methods Med Res. 2002;11(I):49-67.
25. Brant R. Assessing proportionality in the proportional odds model for ordered logistic regression. Biometrics. 1990;46: 1171-8.
26. Roberts MJ, Campbell JL, Abel GA, al et. Understanding high and low patient experience scores in primary care: analysis of patients' survey data for general practices and individual doctors. BMJ. 2014;349:g6034.
27. Ashworth M, Armstrong D. The relationship between general practice characteristics and quality of care: a national survey of quality indicators used in the UK Quality and Outcomes Framework, 2004–5. BMC Fam Pract. 2006;7:68.
28. Murante AM, Seghieri C, Vainieri M, Schäfer WLA. Patient-perceived responsiveness of primary care systems across Europe and the relationship with the health expenditure and remuneration systems of primary care doctors. Soc Sci Med. 2017;185:139-47.
29. McLean G, Guthrie B, Mercer SW, Watt GC. General practice funding underpins the persistence of the inverse care law: cross-sectional study in Scotland. Br J Gen Pract. 2015; 65(641):799-805.
30. Levene L, Baker R, Wilson A, Walker N, Boomla K, Bankart M. Population health needs as predictors of variations in NHS practice payments: a cross-sectional study of English general practices in 2013–2014 and 2014–2015. Br J Gen Pract. 2016;67(654):e10-e9.
31. Mukhtar TK, Bankhead C, Stevens S, Perera R, Holt TA, Salisbury C, et al. Factors associated with consultation rates in general practice in England, 2013–2014: a cross-sectional study. Br J Gen Pract 2018;68(670):e370-e7.
32. Lyratzopoulos G, Elliott M, Barbiere JM, al et. Understanding ethnic and other socio-demographic differences in patient experience of primary care: evidence from the English General Practice Patient Survey. BMJ Qual Saf. 2012;21(1):21-9.
33. Bonciani M, Schäfer W, Barsanti S, Heinemann S, Groenewegen PP. The benefits of co-location in primary care practices: the perspectives of general practitioners and patients in 34 countries. BMC Health Serv Res. 2018 18(1):132.
34. Gosden T, Forland F, Kristiansen IS, Sutton M, Leese B, Giuffrida A, et al. Impact of payment method on behaviour of primary care physicians: a systematic review. J Health Serv Res Policy. 2001;6(1):44-55.
35. NHS England. The NHS Long Term Plan. NHS England, 2019.
36. Care Quality Commission. The five key questions we ask: Care Quality Commission; 2016 [12th April 2017]. Available from: http://www.cqc.org.uk/content/five-key-questions-we-ask.

For peer review only - http://bmjopen.bmj.com/site/about/guidelines.xhtml

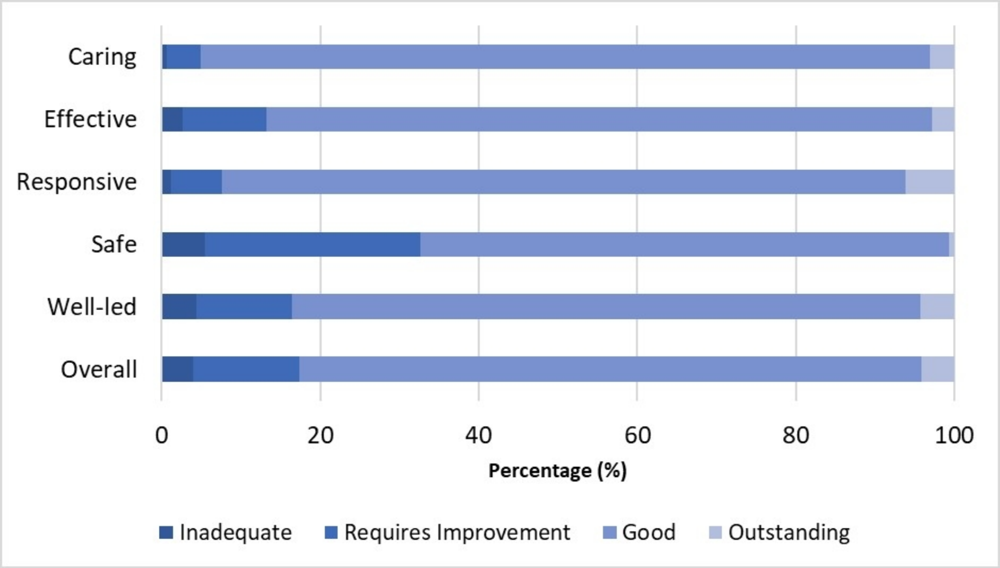

Figure 1: Distribution of CQC ratings by each domain: Distribution of CQC ratings by each domain

159x90mm (150 x 150 DPI)

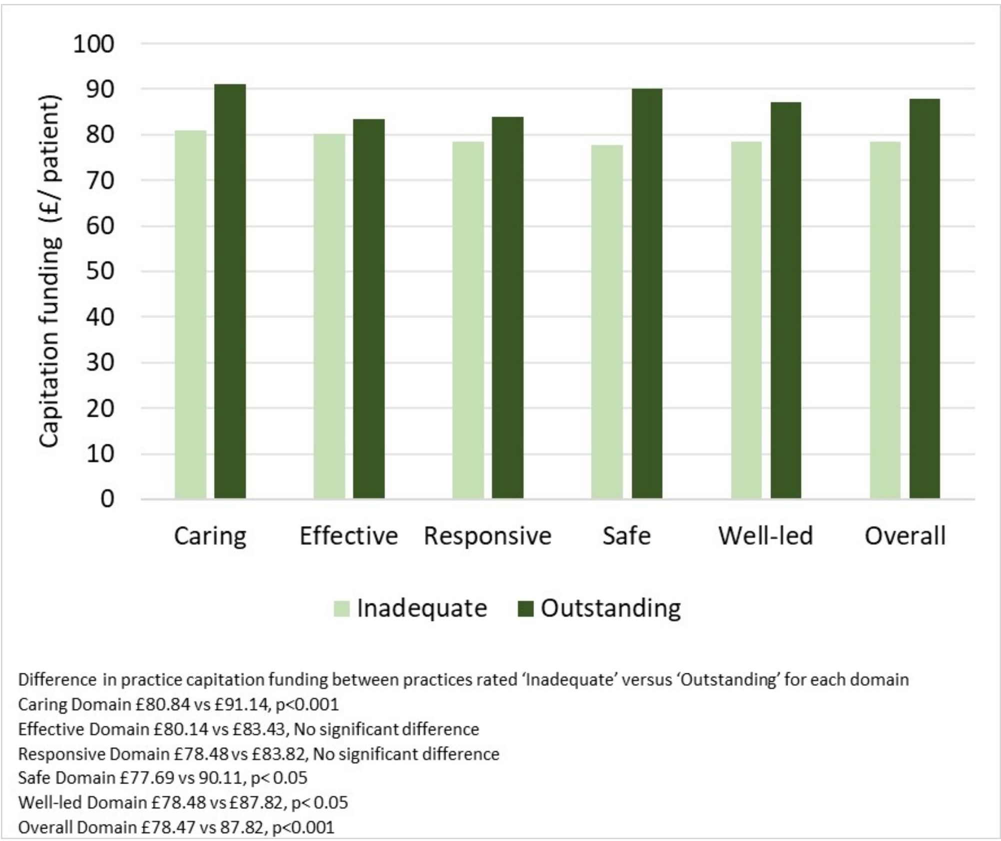

Figure 2: Practice capitation funding by overall practice rating: 'Inadequate' versus 'Outstanding'

150x125mm (150 x 150 DPI)

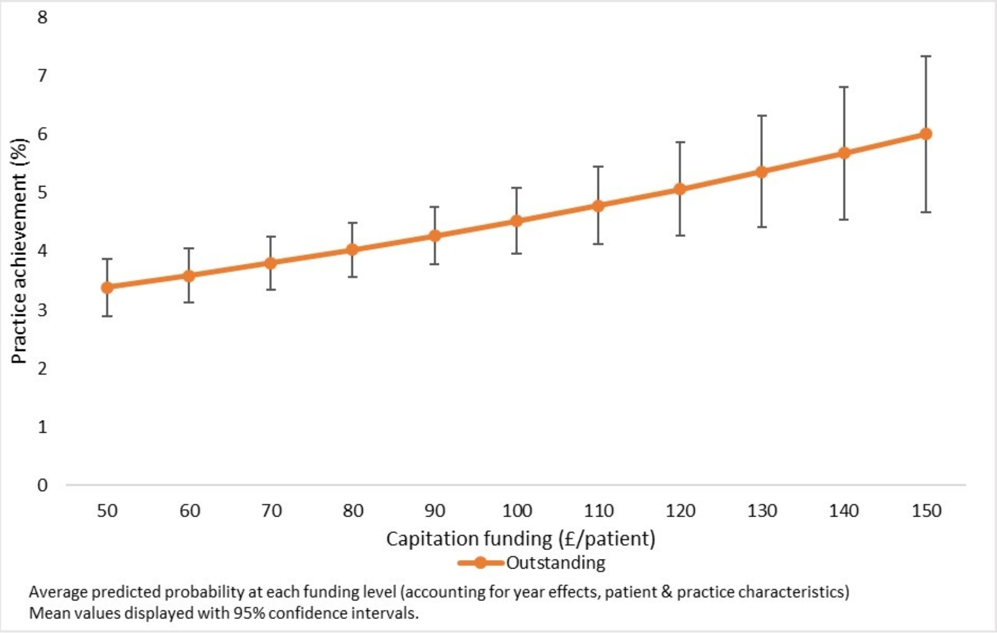

Figure 3: Estimated probability of overall practice rating of 'Outstanding' at various levels of capitation funding per registered patient

159x100mm (150 x 150 DPI)

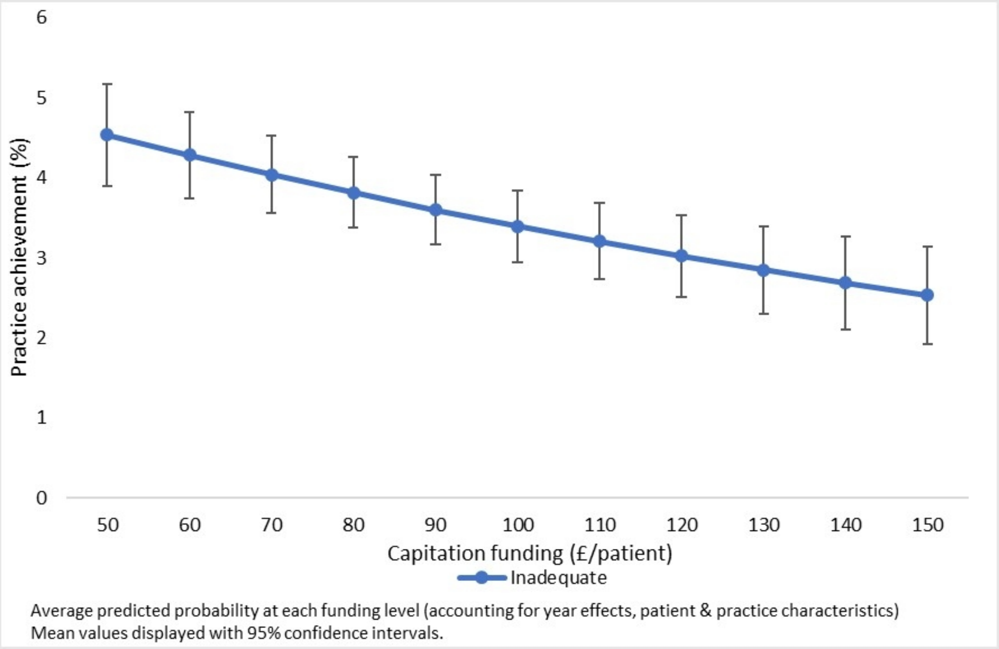

Figure 4: Estimated probability of overall practice rating of 'Inadequate' at various levels of capitation funding per registered patient

150x98mm (150 x 150 DPI)

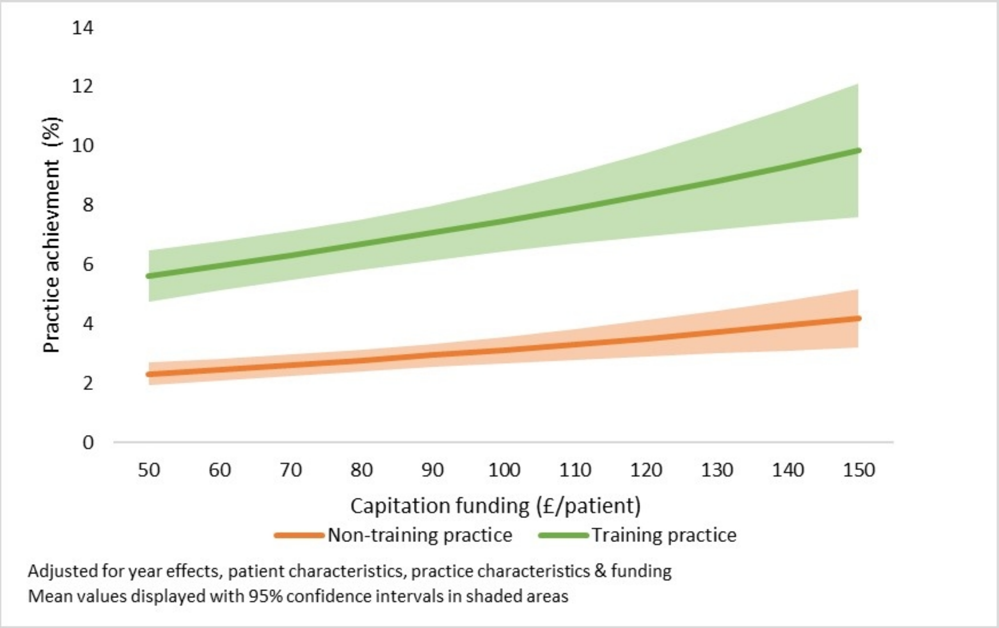

Figure 5: Estimated probability of 'Outstanding' overall practice CQC rating: training versus non-training practices

147x93mm (150 x 150 DPI)

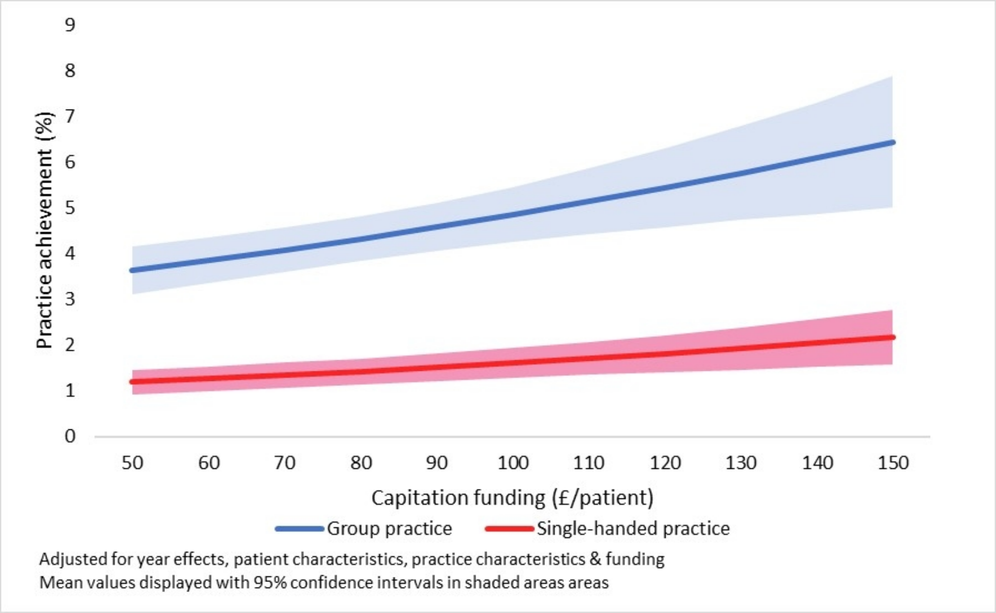

Figure 6: Estimated probability of 'Outstanding' overall practice CQC rating: single-handed versus group practices

152x93mm (150 x 150 DPI)

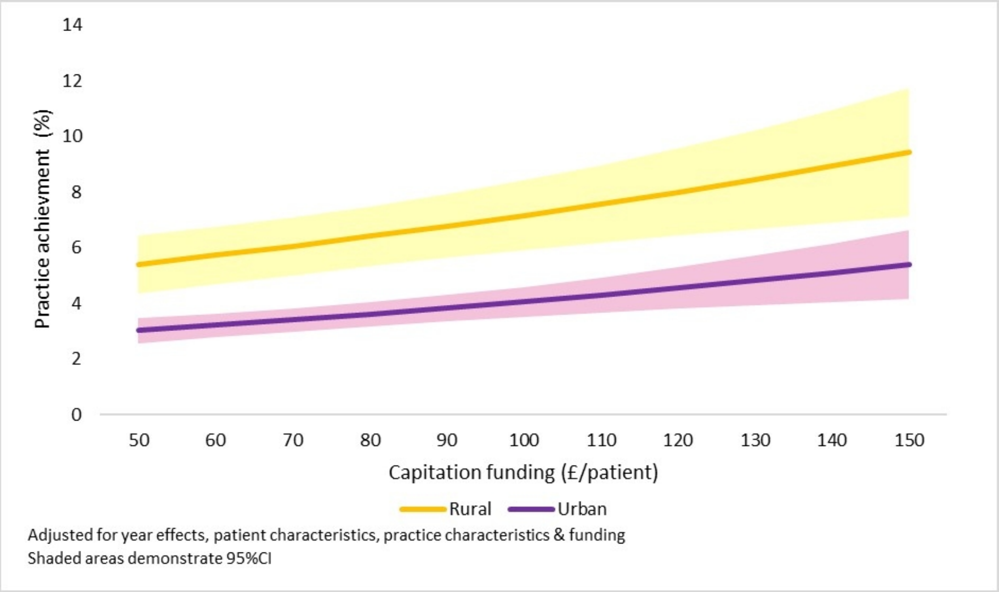

Figure 7: Estimated probability of 'Outstanding' overall practice CQC rating: rural and urban practices

159x94mm (150 x 150 DPI)

STROBE Statement—checklist of items that should be included in reports of observational studies

	Item No.	Recommendation	Page No.	Relevant text from manuscript
**Title and abstract**	1	(*a*) Indicate the study's design with a commonly used term in the title or the abstract	1	"Cross-sectional study"
		(*b*) Provide in the abstract an informative and balanced summary of what was done and what was found	1	Summarised in Abstract: Design, Participants, Main outcome measures, Results
**Introduction**				
Background/rationale	2	Explain the scientific background and rationale for the investigation being reported	2-3	The link between measured dimensions of quality and primary care funding data
Objectives	3	State specific objectives, including any prespecified hypotheses	3	"we assess the relationship of practice capitation funding with overall CQC ratings and with the individual CQC domains"
**Methods**				
Study design	4	Present key elements of study design early in the paper	5	Presented under'Sample'.
Setting	5	Describe the setting, locations, and relevant dates, including periods of recruitment, exposure, follow-up, and data collection	3	Presented under 'Data Sources'
Participants	6	(*a*) *Cohort study*—Give the eligibility criteria, and the sources and methods of selection of participants. Describe methods of follow-up<br>*Case-control study*—Give the eligibility criteria, and the sources and methods of case ascertainment and control selection. Give the rationale for the choice of cases and controls<br>*Cross-sectional study*—Give the eligibility criteria, and the sources and methods of selection of participants	5	Cross-sectional study: general practice eligibility criteria summarised under 'Sample'
		(*b*) *Cohort study*—For matched studies, give matching criteria and number of exposed and unexposed<br>*Case-control study*—For matched studies, give matching criteria and the number of controls per case		

For peer review only

Variables	7	Clearly define all outcomes, exposures, predictors, potential confounders, and effect modifiers. Give diagnostic criteria, if applicable	4/5	Practice variables summarised under the heading 'practice data'; funding variables summarised under the heading 'practice funding data'
Data sources/ measurement	8*	For each variable of interest, give sources of data and details of methods of assessment (measurement). Describe comparability of assessment methods if there is more than one group	3	All sources of data listed under the heading, 'Data Sources'
Bias	9	Describe any efforts to address potential sources of bias	4	The large no. of variables included in our regression model are designed to maximise the explanatory power of the model
Study size	10	Explain how the study size was arrived at	5	Included practices represent ALL practices, minus exclusions based on predetermined exclusion criteria. Only lack of data availability restricted the sample size.

Continued on next page

For peer review only

Quantitative variables	11	Explain how quantitative variables were handled in the analyses. If applicable, describe which groupings were chosen and why	5/6	Quantitative variables analysed according to description in section headed, 'Data Analysis'.
Statistical methods	12	(*a*) Describe all statistical methods, including those used to control for confounding	5/6	Regression modelling – see Data Analysis
		(*b*) Describe any methods used to examine subgroups and interactions	6	Sub-groups such as 'outstanding' or 'inadequate' practices identified
		(*c*) Explain how missing data were addressed	5/6	Practices excluded if missing data
		(*d*) *Cohort study*—If applicable, explain how loss to follow-up was addressed<br>*Case-control study*—If applicable, explain how matching of cases and controls was addressed<br>*Cross-sectional study*—If applicable, describe analytical methods taking account of sampling strategy	n/a	The study was not a sample
		(*e*) Describe any sensitivity analyses	8	See heading 'Sensitivity analyses'
**Results**				
Participants	13*	(a) Report numbers of individuals at each stage of study—eg numbers potentially eligible, examined for eligibility, confirmed eligible, included in the study, completing follow-up, and analysed	5	Summarised under 'sample'. 7310 practices in England; 54 excluded.
		(b) Give reasons for non-participation at each stage	n/a	n/a (exclusion criteria stated above)
		(c) Consider use of a flow diagram	n/a	Exclusion criteria summarised on pg5 without use of a Flow diagram
Descriptive data	14*	(a) Give characteristics of study participants (eg demographic, clinical, social) and information on exposures and potential confounders	13	Table 2 summarises, 'Characteristics of general practices and their populations in England'
		(b) Indicate number of participants with missing data for each variable of interest	n/a	Sample only included those with available data
		(c) *Cohort study*—Summarise follow-up time (eg, average and total amount)	n/a	
Outcome data	15*	*Cohort study*—Report numbers of outcome events or summary measures over time	n/a	
		Case-control study—Report numbers in each exposure category, or summary measures of exposure	n/a	
		Cross-sectional study—Report numbers of outcome events or summary measures	13	Summary measures in Table 2
Main results	16	(*a*) Give unadjusted estimates and, if applicable, confounder-adjusted estimates and their precision (eg, 95% confidence interval). Make clear which confounders were adjusted for and why they were included	14	Table 4 summarises 'unadjusted' and three levels of 'adjusted' analyses

For peer review only - http://bmjopen.bmj.com/site/about/guidelines.xhtml

		15	Confounders adjusted for are listed in Table 5
	(*b*) Report category boundaries when continuous variables were categorized	n/a	No categorisation of continual variables
	(*c*) If relevant, consider translating estimates of relative risk into absolute risk for a meaningful time period	n/a	Absolute financial values are presented in the findings.

Continued on next page

For peer review only

For peer review only - http://bmjopen.bmj.com/site/about/guidelines.xhtml

Other analyses	17	Report other analyses done—eg analyses of subgroups and interactions, and sensitivity analyses	8	Sensitivity analyses summarised
**Discussion**				
Key results	18	Summarise key results with reference to study objectives	9	Key finding summarised at opening of Discussion: 'This study has demonstrated that higher capitation funding is associated with significantly higher overall practice quality ratings and ratings across all individual domains.'
Limitations	19	Discuss limitations of the study, taking into account sources of potential bias or imprecision. Discuss both direction and magnitude of any potential bias	10	Discussed under heading, 'Strengths and Weaknesses of the study'
Interpretation	20	Give a cautious overall interpretation of results considering objectives, limitations, multiplicity of analyses, results from similar studies, and other relevant evidence	10/11	Cautious interpretation summarised with reference to the literature
Generalisability	21	Discuss the generalisability (external validity) of the study results	11	Summarised under, 'Implications for policy and practice'
**Other information**				
Funding	22	Give the source of funding and the role of the funders for the present study and, if applicable, for the original study on which the present article is based	In submission details	First author is funded as Doctoral Research Fellow (DRF) by NIHR

*Give information separately for cases and controls in case-control studies and, if applicable, for exposed and unexposed groups in cohort and cross-sectional studies.

Note: An Explanation and Elaboration article discusses each checklist item and gives methodological background and published examples of transparent reporting. The STROBE checklist is best used in conjunction with this article (freely available on the Web sites of PLoS Medicine at http://www.plosmedicine.org/, Annals of Internal Medicine at http://www.annals.org/, and Epidemiology at http://www.epidem.com/). Information on the STROBE Initiative is available at www.strobe-statement.org.

For peer review only

The relationship between general practice capitation funding and the quality of primary care in England: a cross-sectional, three-year study

Journal:	*BMJ Open*
Manuscript ID	bmjopen-2019-030624.R2
Article Type:	Original research
Date Submitted by the Author:	27-Sep-2019
Complete List of Authors:	L'Esperance, Veline; King's College London, UK, School of Population Health and Environmental Sciences<br>Gravelle, Hugh; University of York, Centre for Health Economcs<br>Schofield, Peter; King's College London, UK, School of Population Health and Environmental Sciences<br>Santos, Rita; University of York, Centre for Health Economics<br>Ashworth, Mark; King's College London, UK, School of Population Health and Environmental Sciences
**Primary Subject Heading**:	General practice / Family practice
Secondary Subject Heading:	Health economics, Health policy, Health services research
Keywords:	PRIMARY CARE, Quality in health care < HEALTH SERVICES ADMINISTRATION & MANAGEMENT, HEALTH ECONOMICS, Health inequalities, primary care funding, general practice

Title:

The relationship between general practice capitation funding and the quality of primary care in England: a cross-sectional, three-year study

Authors:

Veline L'Esperance[1], Hugh Gravelle[2], Peter Schofield[3], Rita Santos[4], Mark Ashworth[5]

1. Doctoral Research Fellow, School of Population Health & Environmental Sciences, Kings College London, London, UK
2. Professor of Economics, Centre for Health Economics, University of York, York, UK
3. Wolfson Lecturer, School of Population Health & Environmental Sciences, Kings College London, London, UK
4. Research Fellow, Centre for Health Economics, University of York, York, UK
5. Reader in Primary Care, School of Population Health & Environmental Sciences, Kings College London, London, UK

Corresponding author email: veline.lesperance@kcl.ac.uk

Abstract:

Objective To explore the relationship between general practice capitation funding and quality ratings based on general practice inspections.

Design Cross-sectional study pooling three years of primary care administrative data.

Setting UK primary care.

Participants 7310 practices (95% of all practices) in England which underwent Care Quality Commission (CQC) inspections between November 2014 and December 2017.

Main outcome measures CQC ratings. Ordered logistic regression methods were used to predict the relationship between practice capitation funding and CQC ratings in each of five domains of quality: caring, effective, responsive, safe and well-led, together with an overall practice rating.

Results Higher capitation funding per patient was significantly associated with higher CQC ratings across all five quality domains; caring (odds ratio [OR] 1.14, 95% confidence interval [CI]: 1.04 to 1.23); effective (OR 1.08, 95% CI: 1.00 to 1.16); responsive (OR 1.09, 95%CI:

For peer review only - http://bmjopen.bmj.com/site/about/guidelines.xhtml

1.02 to 1.17); safe (OR 1.11, 95% CI: 1.05 to 1.18); well-led (OR 1.13, 95% CI: 1.06 to 1.20); and overall rating (OR 1.13, 95% CI: 1.06 to 1.19).

Conclusion: Higher capitation funding was consistently associated with higher ratings across all CQC domains and in the overall practice rating. This study suggests that measured dimensions of the quality of care are related to the underlying capitation funding allocated to each general practice, implying that additional capitation funding may be associated with higher levels of primary care quality.

Strengths and limitations

- A cross-sectional study covering three years of primary care data
- The definition of primary care quality used in this study was multidimensional, based on inspection findings and covering patient safety, patient experience, clinical effectiveness.
- The association between the achievement of quality ratings and practice capitation funding was explored, adjusted for known confounders
- Although based on a near complete sample of general practices in England, bias may have been introduced by data coding and recording errors
- Longer term and prospective studies are required to strengthen causal inferences

For peer review only - http://bmjopen.bmj.com/site/about/guidelines.xhtml

Introduction

Improving the quality of care is a major focus of UK government health policy(1). High quality health care has three main components: clinical achievement, patient experience and patient safety(2). There is wide variation between general practices in the achievement of clinical care quality indicators and patient reported satisfaction(3, 4).

It is important to understand whether variations in the quality of care provided across practices are related to variations in their funding. Healthcare quality regulation in England is currently undertaken by the Care Quality Commission, focuses on outcomes for patients and has a wide range of enforcement powers, including closure and deregistration of services, if essential standards are not met.(5)

Studies of the relationship between quality and funding in English general practices have largely focussed on the Quality and Outcomes Framework (QOF) which rewards practices for higher quality care, as defined by the achievement of clinical process and outcome targets. The QOF has had limited impact on reducing secondary care costs(6) or improving primary care performance(7, 8). In terms of financial incentivisation, the QOF accounted for approximately 7.8% of funding received by general practices in England in 2016(9). In contrast, capitation payments represent the largest proportion of funding to general practice (54% in 2016) and are related to the number of registered patients in each practice(9), adjusted for factors thought to increase the demand on primary care services(10). Other components of general practice funding include additional payments for postgraduate training, the provision of additional clinical services ('enhanced services') and various

For peer review only - http://bmjopen.bmj.com/site/about/guidelines.xhtml

reimbursements to cover the costs of premises, computers and for some practices, dispensing medication(11).

Greater capitation spending on general practices has been found to be associated with reductions in secondary care usage and costs, and increased patient satisfaction(12). Studies have also shown that leadership within the practice organisation plays a key role in the delivery of high quality care(13). Until recently, nationally derived metrics of inspection-based primary care quality were unavailable. Since October 2014 all general practices have been subjected to inspections by the Care Quality Commission (CQC) (14), (5). The CQC reports on the extent to which practices are caring, effective, responsive to the needs of patients, safe, and well-led(5, 15) and also combines these five domains to produce an overall practice rating. These five domains incorporate components of clinical achievement, patient experience and patient safety(2). In this study, we assess the relationship of practice capitation funding with overall CQC ratings and with the individual CQC domains. We aimed to examine the relationship between practice funding and the quality of care as determined by inspection-based quality assessment. Analysis of total practice funding would have introduced confounding through inclusion of quality-related payments. We therefore used capitation funding as our measure of practice funding since this financial indicator is independent of financial rewards associated with quality achievement such as the QOF and other national and local incentive schemes.

Methods

Data sources

We linked practice-level data on NHS payments to general practice identifiers(16), CQC inspection ratings(15), NHS administrative datasets, General and Personal Medical Services

For peer review only - http://bmjopen.bmj.com/site/about/guidelines.xhtml

Statistics (17), and small area Census and socio-economic data from Neighbourhood Statistics(18).

Care Quality Commission Ratings

CQC ratings are based on publicly available data (such as QOF and General Practice Patient Survey(19)), practice inspections, interviews with patients and staff, complaints, clinical record reviews, reviews of practice documents and policies(15). We used CQC ratings for practices with completed CQC reports first inspected between November 2014 and December 2017 (n = 7310, 95% of all practices). Practice ratings were obtained from the CQC; these data are publicly available on request. For practices which required reinspection only the first inspection score was included in the analysis. The five domains of quality described by CQC inspections are summarised in Table 1; each is rated on a 4-point scale.

Practice data

Data for all general practices in England were obtained from the General and Personal Medical Services database, for 2014/15, 2015/16 and 2016/17 financial years (17). These data are publicly available from NHS Digital. Our use of practice based demographic data followed a previously used methodology(20). Patient characteristics included the proportion of patients aged 0 to 4 years, proportion of patients aged 75 years or older and proportion of nursing home patients. Deprivation data for each general practice was attributed as the mean of the Index of Multiple Deprivation (IMD) 2015(18) weighted by the proportion of practice patients resident in each Lower Layer Super Output area (LLSOA). Neighbourhood ethnicity (proportion Asian or black) derived from the 2011 national census, was attributed to practices weighted by the proportion of the practice population in each LLSOA(21). The following

For peer review only - http://bmjopen.bmj.com/site/about/guidelines.xhtml

practice characteristics were included: region (North, Midlands, London and South), contract type (General Medical Services or Personal Medical Services), minimum distance from an acute hospital, dispensing status (whether the practice dispensed as well as prescribed medication), singlehanded practice status (singlehanded practices have ≤1.0 FTE GP; group practices have >1.0 FTE GPs) and training practice status. We did not include practice staffing (GPs, nurses, other staff) as explanatory variables in the model because staffing is likely to be directly affected by practice capitation funding and so inclusion of these variable may lead to an underestimate of the full effect of capitation funding. Moreover, a major change in the way in which staffing data was collected in 2015/16 would have resulted in a large reduction in observation number.

Practice capitation

Practice capitation funding depends on the total number of practice patients adjusted to reflect factors affecting GP workload (age, gender, patients in nursing and residential homes, small area measures of morbidity), rurality and an index of local staff costs which affect the cost of providing services(10). Data was available for the financial years 2014/15, 2015/16 and 2016/7(22). We use the mean capitation payment per patient for the year prior to inspection and the year in which the practice was inspected.

Sample

We linked inspected practices (n=7310) with funding data for their year of inspection. We excluded atypical practices with ≤750 registered patients (n=10) or ≤500 patients per FTE GP (n= 8) following a previously used method(23). Practices with recorded negative (n= 2) or zero funding (n = 52) were excluded. The final analysis sample consisted of 7238 practices.

For peer review only - http://bmjopen.bmj.com/site/about/guidelines.xhtml

Data Analysis

Analysis was conducted at GP practice level. Since the CQC rating outcomes are ordered categories we used ordered logistic regression to model the relationship between funding and the practice CQC ratings(24). Separate models were estimated for each domain.

The key explanatory variable was capitation funding per patient (measured in standard deviation units). We also include patient and practice characteristic covariates, thereby reducing the risk of bias from the omission of variables which might affect the CQC rating and are correlated with practice capitation funding. The regression models included year effects to allow for inspection year and annual general practice funding uplifts. We accounted for local area effects by adjusting for clustering at Clinical Commissioning Group (CCG) level. Multicollinearity was tested for by calculating the Variance Inflation Factor (VIF) and variables with a value for VIF>5 were excluded. The proportional odds assumption of the ordered logit model was also tested(25). We report the odds ratio from the estimated models.

We calculated the average marginal effects of funding on the predicted probabilities of achieving overall ratings of "outstanding" and "inadequate" for all practices. We also compared the predicted probabilities of an "outstanding" overall rating at different practice capitation funding levels for training versus non-training practices, single-handed versus multi-handed practices, and rural versus urban practices. STATA 14 (StataCorp, College Station, TX) was used for all statistical analyses.

Patient involvement

For peer review only - http://bmjopen.bmj.com/site/about/guidelines.xhtml

Funding for this study included funding of a dedicated patient involvement group. Patients were involved in developing plans for the study design, approving the outcome measures and commenting on the potential impact of outcomes. A lay summary was also provided.

Results

Summary statistics for the main characteristics of the general practices are provided in Table 2. Mean practice capitation funding per registered patient increased from £77.49 in 2014/5 to £83.17 in 2016/7 (Table 3). The mean capitation funding per patient across the CQC inspection period was £79.48. The standard deviation of the mean capitation funding per patient was £22.00.

The distribution of practice ratings across each quality domain is shown in Figure 1. A total of 79% (n = 5774) of practices achieved an overall rating of 'Good', while only 4% (294) achieved an overall rating of 'Outstanding'. 'Inadequate' ratings varied across the domains, from 1% (caring domain) to 6% (safety domain) and 4% (overall).

Figure 2 shows the difference in capitation funding for practices with the lowest quality rating compared to those with the highest quality rating. In each domain, 'Inadequate' practices received less capitation funding. Using an independent group t-test this difference was found to be significant for three domains (caring, safe, well-led) and for the overall practice rating.

Table 4 reports the odds ratios on capitation funding per patient estimated from four regression models of overall practice CQC rating. The odds ratio on capitation funding per patient is reported in standard deviation (SD units). In the first model capitation funding is the only explanatory variable (unadjusted model); remaining models are adjusted for

For peer review only - http://bmjopen.bmj.com/site/about/guidelines.xhtml

inclusion of successive additional explanatory variables: year effects, patient characteristics, and practice characteristics. The unadjusted model shows an association between higher capitation funding and higher overall CQC ratings with an odds ratio (OR) of 1.09 (95% confidence interval (CI): 1.03 to 1.15). Allowing for the year of inspection increased the OR slightly to 1.10 (95%CI: 1.04 to 1.16). Additional allowance for patient characteristics (OR 1.13; 95%CI: 1.06 to 1.19) and practice characteristics (OR 1.13; 95%CI: 1.06 to 1.19) further increased the odds ratio. The number of observations in Table 4 fell from 7,168 to 7,045 because of missing data. Very similar changes in odds ratios across the models were observed when all models were restricted to equal sample sizes. A likelihood ratio test demonstrated that the addition of patient and practice variables create a statistically significant improvement in model fit, confirming that higher odds ratios were associated with the addition of model variables, rather than to a change in sample size.

The final adjusted model indicates that for a one standard deviation increase in capitation funding, the odds of achieving an outstanding CQC rating are 1.13 times greater, given that other variables are held constant. We have also shown the estimated changes in the probabilities of achieving ‘inadequate’ and ‘outstanding’ CQC ratings implied by this model in Figures 3 to 7.

Table 5 reports odds ratios for all the explanatory variables in the overall practice quality rating model (Model 4, Table 4). In addition to higher practice capitation funding, rural practice and training practice status were significantly associated with higher overall practice ratings. For example, the adjusted odds ratio of a training practice achieving an ‘outstanding’

For peer review only - http://bmjopen.bmj.com/site/about/guidelines.xhtml

CQC rating is 2.30 times greater than for a non-training practice. Conversely, for singlehanded practices the odds of achieving an ‘outstanding’ rating is 0.53 times that for group practices.

The odds ratios for capitation funding per patient from the full models for each CQC domain are shown in Table 6. Higher capitation funding was significantly associated with higher CQC ratings across all five quality domains.

We used the results from the ordered logistic regression models with the full set of explanatory variables to calculate the probability of achieving an overall practice rating of ‘Outstanding’ or ‘Inadequate’ at different levels of capitation funding. Figure 3 shows the average predicted probability of achieving an ‘Outstanding’ rating for a range of per capita funding levels. The probabilities are the average of the estimated probabilities for each practice calculated at each funding level using actual values of the practice non-funding characteristics (year effects, patient characteristics and practice characteristics). Figure 4 shows the average predicted probability of achieving an ‘Inadequate’ practice rating. Higher capitation funding was associated with reduced probability of achieving an ‘Inadequate’ rating and increased probability of an ‘Outstanding’ quality rating. At capitation payments above £100 per patient, practices have a greater probability of being rated as “Outstanding” rather than “Inadequate”.

We also compared the probability of achieving an ‘Outstanding’ rating at different levels of practice capitation funding for training versus non-training practices (Figure 5), for single-handed versus group practices (Figure 6), and for rural versus urban practices (Figure 7). At all levels of funding, the probability of achieving an ‘Outstanding’ rating is higher for training practices than non-training practices, for group practices than single handed practices, and

For peer review only - http://bmjopen.bmj.com/site/about/guidelines.xhtml

rural practices than urban practices. In all cases higher capitation funding is associated with higher probabilities of an ’Outstanding’ rating.

Sensitivity Analyses

The Brant test(25) assesses the proportional odds assumption that the distance between each category is equivalent. Four of the variables included in our model (region, proportion of patients aged 0-4 years, contract type and single-hander status) did not meet the assumption of proportionality of the odds ratios. However, our variable of interest, capitation funding per patient, did not violate the proportional odds assumption. A partial proportional odds model excluding these four variables, estimated by generalised ordered logistic regression, yielded similar results to our main model: higher capitation funding was significantly associated with increase probability of achieving an ‘Outstanding’ rating (OR 1.14, 95% CI: 1.04 to 1.25).

Discussion

This study has demonstrated that higher capitation funding is associated with significantly higher overall practice quality ratings and ratings across all individual domains.

Practice characteristics such as post-graduate training practice and group practice status were also associated with higher quality ratings, representing primary care structures which support higher quality of care. However, some factors related to the registered practice population, such as urban location, social deprivation and larger proportions of ethnic minority patients were negatively associated with the practice quality of care rating. Many of these factors are already known to be negatively associated with reported patient satisfaction (26) and QOF achievement (27). Including them in the model led to a stronger association of practice capitation funding with practice quality rating. The likely reason for this is that

For peer review only - http://bmjopen.bmj.com/site/about/guidelines.xhtml

practice capitation funding is positively correlated with patient characteristics which have negative effects on the quality rating. Thus, including these patient characteristics in the model removes a source of bias from omitted variables which would otherwise tend to underestimate the positive association of funding with the quality rating.

Strengths and weaknesses of the study

This is the first study to explore the relationship between practice-level capitation funding and practice quality as measured by CQC ratings. The findings are based on a near complete sample of general practices across England. Using data linkages from a wide range of sources, and multilevel statistical models, this study has been able to demonstrate the independent effects of practice funding and practice characteristics on quality ratings, which might otherwise be confounded in single-level analyses. A variety of sensitivity analyses have confirmed the robustness of the ordered logistic regression modelling.

However, there are several limitations. Routinely collected data are subject to coding and recording errors. As with all observational studies, significant associations, even if large, may not be causal. Although a wide range of potential confounders were included in the models, confounding by omitted variables cannot be excluded.

Comparison with existing literature

These findings complement those of a previous study which found that increased general practice capitation funding was associated with reduced emergency hospital admissions and Accident and Emergency attendances(12).

For peer review only - http://bmjopen.bmj.com/site/about/guidelines.xhtml

For peer review only

In a country level European analysis it was found that systems relying on capitation funding were more responsive than those based on fee for service or mixed payment(28). However, analysis of Scottish general practices suggests that capitation funding may contribute to the persistence of the inverse care law with deprived areas experiencing lower quality of care, as defined by inspection ratings(29). Consistent with our study, others have found that GP practice funding is negatively correlated with healthcare need predictors such as deprivation and non-white ethnicity(30). Previous studies have also demonstrated that greater GP workload may be associated with higher levels of social deprivation and with a higher proportion of Asian patients(31). Similarly, practices with a greater proportion of ethnically diverse patients reported worse patient experience(32). Our work is also consistent with a recent study which demonstrated that GPs co-located with other GPs and professionals had improved outcomes compared with single-handed GP practices such as broader provision of technical procedures, wider coordination with secondary care and increased collaboration among different providers (33).

Our study was based on funding data for general practices but was unable to study the relationship between quality ratings and individual GP income. However, values for overall 'profit' per practice are expected to become available in due course. Other studies have confirmed that incentives based on personal income may influence both quality achievement and productivity(34).

Implications for policy and practice

This work provides further evidence of the association between general practice capitation funding and the quality of primary care. A causal association is plausible and supports the

For peer review only - http://bmjopen.bmj.com/site/about/guidelines.xhtml

argument that increased quality and safety of patient care may be achieved through additional investment. The recently published NHS Long Term Plan(35) outlines proposals to offer increases in capitation payment together with an emphasis on inter-practice cooperation through the formation of primary care networks. Both factors are likely to influence the relationship between funding and the quality of primary care and will require further study. Our findings suggest that revisions to the primary care capitation formula are necessary to ensure that additional funding is provided in urban areas of high deprivation and ethnic minority populations in order to address quality of care inequalities.

Unanswered questions and future research

Future research could extend similar analyses to subsequent 3-year cycles of quality inspection. A longitudinal approach, relating changes in funding to changes in outcomes, is likely to provide more accurate estimates of the effect of funding. Complementary qualitative analysis is likely to provide insight into mechanisms underlying the link between better funded practices and higher quality rating achievement.

Conclusion

Higher capitation funding was consistently associated with higher overall and domain quality ratings yielded by CQC inspections. This study suggests that measured and inspected dimensions of the quality of care are related to the underlying funding allocated to each general practice, implying that additional funding may be associated with higher levels of primary care quality.

What is already known on this topic

- Few studies on the relationship between capitation funding and quality

For peer review only - http://bmjopen.bmj.com/site/about/guidelines.xhtml

- In England, the QOF has had limited impact on reducing healthcare costs and improving primary care performance

What this study adds

- Higher capitation funding is associated with increased quality ratings across all domains
- Since the NHS Long Term Plan calls for increased funding to primary care it is important to understand how and where this funding should be directed
- Our findings imply that further primary care funding is particularly needed in areas of high deprivation and ethnic minority populations where funding may be insufficient to support high quality of care.

For peer review only

For peer review only - http://bmjopen.bmj.com/site/about/guidelines.xhtml

Contributors: VL, HG, PS, RS and MA contributed to the idea and design of the study. VL and PS led on data analysis with statistical advice from HG, RS and MA. VL produced the first draft of the paper; all co-authors contributed and approved the final draft. VL is the guarantor. The corresponding author attests that all listed authors meet authorship criteria and that no others meeting the criteria have been omitted.

Funding: This work was supported by the National Institute for Health Research (NIHR) who funded a Doctoral Research Fellowship for VL (reference, DRF-2017-10-132) and for RS (reference, DRF-2014-07-055). HG and RS were funded by the UK NIHR Policy Research Programme (Policy Research Unit in the Economics of Health and Social Care Systems: Ref 103/0001). The views expressed are those of the authors and not necessarily those of the NHS, the National Institute for Health Research, the Department of Health and Social Care or its arm's length bodies or other UK government departments. The findings presented are independent from the funders who have had no role in study design, data collection, data analysis, data interpretation, or writing of the report.

Competing interests: All authors have completed the ICMJE uniform disclosure form at www.icmje.org/coi_disclosure.pdf and declare: no support from any organisation for the submitted work; no financial relationships with any organisations that might have an interest in the submitted work in the previous three years; no other relationships or activities that could appear to have influenced the submitted work.

Ethical approval: Ethical approval not required for the use of aggregate practice level data as included in this study.

Dissemination declaration: the results have been disseminated to the patient involvement group involved in study design and commenting on potential impact of outcomes.

Data sharing: The reference for each database used in this study is provided.

For peer review only - http://bmjopen.bmj.com/site/about/guidelines.xhtml

Figures and Tables

Table 1: The five key domains for CQC Inspections

Domain	Description
**Safe**	Patients are protected from abuse and avoidable harm
**Effective**	Care, treatment and support achieves good outcomes, helps patients to maintain quality of life and is based on the best available evidence
**Caring**	Staff involve and treat patients with compassion, kindness, dignity and respect
**Responsive**	Services are organised so that they meet patients' needs
**Well-led**	The leadership, management and governance of the organisation make sure it's providing high-quality care that's based around the individual needs, that it encourages learning and innovation, and that it promotes an open and fair culture

Adapted from: CQC. The five key questions we ask(36)

Table 2. Characteristics of general practices and their populations in England

Variable	Mean	(5th, 95th centiles)
Patient adjusted Index of Multiple Deprivation, 2015	24.5	8.2, 46.1
Proportion of patients aged 0 to 4 years (%)	5.9	3.7, 8.8
Proportion of patients aged 75 years or older (%)	7.7	2.6, 12.9
Proportion of patients: nursing home residents (%)	0.5	0, 1.4
Proportion of patients: Asian or black ethnicity (%)	13.1	0.1, 53.1
List size per full-time equivalent (FTE) GP	1950	1066, 3315
List size per FTE non-clinical employed staff	703	392, 1103
List size per FTE nurse	7166	2810, 15507
Minimum distance of practice from acute hospital (km)	3.8	0.4, 11.8
Proportion of practices by rurality (%)		
Urban	85.5	
Rural: hamlet, village, town & fringe	14.5	
Proportion of practices by region (%)		
North	30.3	
Midlands	29.4	
London	18.0	
South	22.3	
Proportion of practices by contract type (%)		
General Medical Services	59.4	
Personal Medical Services	40.6	
Proportion of dispensing practices (%)	14.6	
Proportion of singlehanded practices (%)	13.1	
Proportion of training practices (%)	30.4	

For peer review only - http://bmjopen.bmj.com/site/about/guidelines.xhtml

For peer review only

Table 3: Capitation funding per registered patient for inspected practices

Inspection year	N	Mean capitation funding	(5th, 95th centiles)
2014/15	2232	£77.49	£59.54, £99.99
2015/16	3790	£80.86	£66.57, £101.66
2016/17	1148	£83.71	£67.74, £106.76

Table 4: Association of capitation funding per patient with overall practice CQC rating: unadjusted and adjusted regression models

	Model 1	Model 2	Model 3	Model 4
Capitation funding odds ratio[1]	1.09**	1.10**	1.13***	1.13***
95% CI of odds ratio	1.03, 1.15	1.04, 1.16	1.06, 1.19	1.06, 1.19
Observations	7,168	7,168	7,144	7,045
Models contain				
Year effects	N	Y	Y	Y
Patient characteristics[2]	N	N	Y	Y
Practice characteristics[3]	N	N	N	Y

1. Odds ratios based on Standard Deviation units.
2. Patient adjusted deprivation, proportion of patients aged 0 to 4 years, proportion of patients aged ≥75 years, proportion patients black or Asian ethnicity, proportion of nursing home residents.
3. Region, minimum distance to hospital, contract type, dispensing status, training practice status, singlehanded

*p< 0.05; **p< 0.01; ***p< 0.001

For peer review only

Table 5: Association of capitation funding with overall practice CQC rating: predictor variable in fully adjusted model.

Characteristics	Odds Ratio	95% CI
Capitation funding per patient (SD units)[1]	1.13***	1.06, 1.19
Year 2	0.92	0.80, 1.05
Year 3	0.76**	0.64, 0.91
Deprivation	0.99**	0.98, 0.99
Patients aged 0 to 4 years (proportion)	1.00	0.95, 1.05
Patients aged 75 years or old (proportion)	0.99	0.96, 1.17
Patients in nursing home (proportion)	1.13*	1.02, 1.26
Patients Asian or black ethnicity (proportion)	0.99*	0.99, 1.00
Region: Midlands[§]	0.64***	0.55, 0.76
Region: London[§]	0.56***	0.93, 0.98
Region: South[§]	0.48**	0.40, 0.58
Minimum distance to hospital	1.00	1.00, 1.00
Rurality (Yes/No)	1.50**	1.18, 1.92
Contract type (GMS/PMS)	1.08	0.96, 1.23
Dispensing Practice status (yes/no)	1.10	0.88, 1.38
Singlehanded practice (yes/no)	0.53***	0.44, 0.63
Training Practice status (yes/no)	2.30***	1.99, 2.65
*p< 0.05; **p< 0.01; ***p< 0.001		

[1] Odds ratios based on Standard Deviation units.
[§] Comparator Region: North

For peer review only - http://bmjopen.bmj.com/site/about/guidelines.xhtml

Table 6: Ordered logistic models: effect of capitation funding on each CQC domain rating

Domains	Odds Ratio[1]	95% CI
Caring	1.14**	1.04, 1.23
Effective	1.08*	1.00, 1.16
Responsive	1.09*	1.02, 1.17
Safe	1.11*	1.05, 1.18
Well-led	1.13***	1.06, 1.20
Overall	1.13***	1.06, 1.19

Adjusted for year effects, patient characteristics & practice characteristics &
[1] Odds ratios based on Standard Deviation units.
*$p < 0.05$; **$p < 0.01$; ***$p < 0.001$

Figure 1: Distribution of CQC ratings by each domain

Figure 2: Practice capitation funding by overall practice rating: 'Inadequate' versus 'Outstanding

Figure 3: Estimated probability of overall practice rating of 'Outstanding' at various levels of capitation funding per registered patient

Figure 4: Estimated probability of overall practice rating of 'Inadequate' at various levels of capitation funding per registered patient

Figure 5: Estimated probability of 'Outstanding' overall practice CQC rating: training versus non-training practices

Figure 6: Estimated probability of 'Outstanding' overall practice CQC rating: single-handed versus group practices

Figure 7: Estimated probability of 'Outstanding' overall practice CQC rating: rural and urban practices

For peer review only - http://bmjopen.bmj.com/site/about/guidelines.xhtml

References:

1. Ham C, Berwick D, Dixon J. Improving quality in the English NHS: A strategy for action. London: The Kings Fund, 2016.
2. Department of Health. High Quality Care for All – NHS Next Stage Review Final Report. London: Department of Health, 2008.
3. Campbell S, Steiner A, Robison J, Weber D, Raven A, Roland M. Is the quality of care in general medical practice improving? Results of a longitudinal observational study. Br J Gen Pract 2003;53:298-304.
4. Llanwarne NR, Abel GA, Elliott MN, Paddison CAM, Lyratzopoulos G, Campbell JL, et al. Relationship Between Clinical Quality and Patient Experience: Analysis of Data From the English Quality and Outcomes Framework and the National GP Patient Survey. Annals of Family Medicine. 2013;11(5):467-72.
5. Gillam S, Siriwardena A. Regulation in primary care. Quality in Primary Care 2014;22:57–61.
6. Emmert M, Eijkenaar F, Kemter H, Esslinger AS, Schoffski O. Economic evaluation of pay-for-performance in health care: a systematic review. Eur J Health Econ. 2012;13:755-67.
7. Kontopantelis E., Springate D. A., Ashworth M., Webb R. T., Buchan I. E., Doran T. Investigating the relationship between quality of primary care and premature mortality in England: a spatial whole-population study. BMJ. 2015;350:h904.
8. Campbell SM, Reeves D, Kontopantelis E, Sibbald B, Roland M. Effects of pay for performance on the quality of primary care in England. NEJM. 2009;361:368-78.
9. NHS Digital. NHS Payments to General Practice, England, 2016/17. 2017.
10. British Medical Association. Focus on the global sum allocation formula (Carr-Hill Formula): British Medical Association; 2015 [12th April 2016]. Available from: https://www.google.co.uk/url?sa=t&rct=j&q=&esrc=s&source=web&cd=1&cad=rja&uact=8&ved=0ahUKEwi_sLK47f_OAhWLAMAKHZ2bC8oQFggcMAA&url=https%3A%2F%2Fwww.bma.org.uk%2F-%2Fmedia%2Ffiles%2Fpdfs%2Fpractical%2520advice%2520at%2520work%2Fcontracts%2Fgps%2Ffocus%2520on%2520global%2520sum%2520allocation%2520formula.pdf%3Fla%3Den&usg=AFQjCNEfyEp15cpvpLtT77bIobeHfSmGMQ&sig2=ojUD-oKqav9bcLejniNdtw&bvm=bv.131783435,d.d2s.
11. NHS Digital. General and Personal Medical Services, England: 2005-2015, as at 30 September, Provisional Experimental statistics. NHS Digital, 2016.
12. L'Esperance V, Sutton M, Schofield P, Round T, Malik U, White P, et al. Impact of primary care funding on secondary care utilisation and patient outcomes: a retrospective cross-sectional study of English general practice. Br J Gen Pract. 2017(DOI: https://doi.org/10.3399/bjgp17X693101).
13. Dunham AH, Dunbar JA, Johnson JK, Fuller J, Morgan M, Ford D. What attributions do Australian high-performing general practices make for their success? Applying the clinical microsystems framework: a qualitative study. BMJ Open. 2018;8(4):e020552.
14. Care Quality Commission. The state of care in general practice 2014 to 2017. London: Care Quality Commission, 2017.
15. Care Quality Commission. How CQC monitors, inspects and regulates NHS GP practices. London: Care Quality Commission, 2018.
16. NHS Digital. NHS Payments to General Practice, England: NHS Digital; 2015. Available from: http://digital.nhs.uk/catalogue/PUB18468/nhspaymentsgp-14-15-ann1.xlsx.
17. NHS Digital. General and Personal Medical Services: NHS Digital; 2016. Available from: http://digital.nhs.uk/catalogue/PUB20503.
18. Department for Communities and Local Government. English indices of deprivation 2015. Department for Communities and Local Government 2015.
19. NHS England. The GP Patient Survey. NHS England, 2015 July 2014 Report No.
20. Ashworth M, White P, Jongsma H, Schofield P, Armstrong D. Antibiotic prescribing and patient satisfaction in primary care in England: cross-sectional analysis of national patient survey data and prescribing data. Br J Gen Pract. 2016;66(42).

For peer review only - http://bmjopen.bmj.com/site/about/guidelines.xhtml

21. Office for National Statistics. 2011 Census: Office for National Statistics; 2011 [21st January 2016]. Available from: https://www.ons.gov.uk/census/2011census.
22. NHS Digital. NHS Payments to General Practice, England, 2013-14: Experimental Statistics – Report. Available from: 2015 [5th Sept 2015]. Available from: http://www.hscic.gov.uk/catalogue/PUB16847/nhspaymentsgp-13-14-rep.docx. .
23. Ashworth M, Schofield P, Durbaba S, Ahluwalia S. Patient experience and the role of postgraduate GP training: a cross-sectional analysis of national Patient Survey data in England. Br J Gen Pract. 2014;64(620):168-77.
24. Lall R, Campbell MJ, Walters SJ, Morgan K. A review of ordinal regression models applied on health-related quality of life assessments. Stat Methods Med Res. 2002;11(I):49-67.
25. Brant R. Assessing proportionality in the proportional odds model for ordered logistic regression. Biometrics. 1990;46: 1171-8.
26. Roberts MJ, Campbell JL, Abel GA, al et. Understanding high and low patient experience scores in primary care: analysis of patients' survey data for general practices and individual doctors. BMJ. 2014;349:g6034.
27. Ashworth M, Armstrong D. The relationship between general practice characteristics and quality of care: a national survey of quality indicators used in the UK Quality and Outcomes Framework, 2004–5. BMC Fam Pract. 2006;7:68.
28. Murante AM, Seghieri C, Vainieri M, Schäfer WLA. Patient-perceived responsiveness of primary care systems across Europe and the relationship with the health expenditure and remuneration systems of primary care doctors. Soc Sci Med. 2017;185:139-47.
29. McLean G, Guthrie B, Mercer SW, Watt GC. General practice funding underpins the persistence of the inverse care law: cross-sectional study in Scotland. Br J Gen Pract. 2015; 65(641):799-805.
30. Levene L, Baker R, Wilson A, Walker N, Boomla K, Bankart M. Population health needs as predictors of variations in NHS practice payments: a cross-sectional study of English general practices in 2013–2014 and 2014–2015. Br J Gen Pract. 2016;67(654):e10-e9.
31. Mukhtar TK, Bankhead C, Stevens S, Perera R, Holt TA, Salisbury C, et al. Factors associated with consultation rates in general practice in England, 2013–2014: a cross-sectional study. Br J Gen Pract 2018;68(670):e370-e7.
32. Lyratzopoulos G, Elliott M, Barbiere JM, al et. Understanding ethnic and other socio-demographic differences in patient experience of primary care: evidence from the English General Practice Patient Survey. BMJ Qual Saf. 2012;21(1):21-9.
33. Bonciani M, Schäfer W, Barsanti S, Heinemann S, Groenewegen PP. The benefits of co-location in primary care practices: the perspectives of general practitioners and patients in 34 countries. BMC Health Serv Res. 2018 18(1):132.
34. Gosden T, Forland F, Kristiansen IS, Sutton M, Leese B, Giuffrida A, et al. Impact of payment method on behaviour of primary care physicians: a systematic review. J Health Serv Res Policy. 2001;6(1):44-55.
35. NHS England. The NHS Long Term Plan. NHS England, 2019.
36. Care Quality Commission. The five key questions we ask: Care Quality Commission; 2016 [12th April 2017]. Available from: http://www.cqc.org.uk/content/five-key-questions-we-ask.

For peer review only - http://bmjopen.bmj.com/site/about/guidelines.xhtml

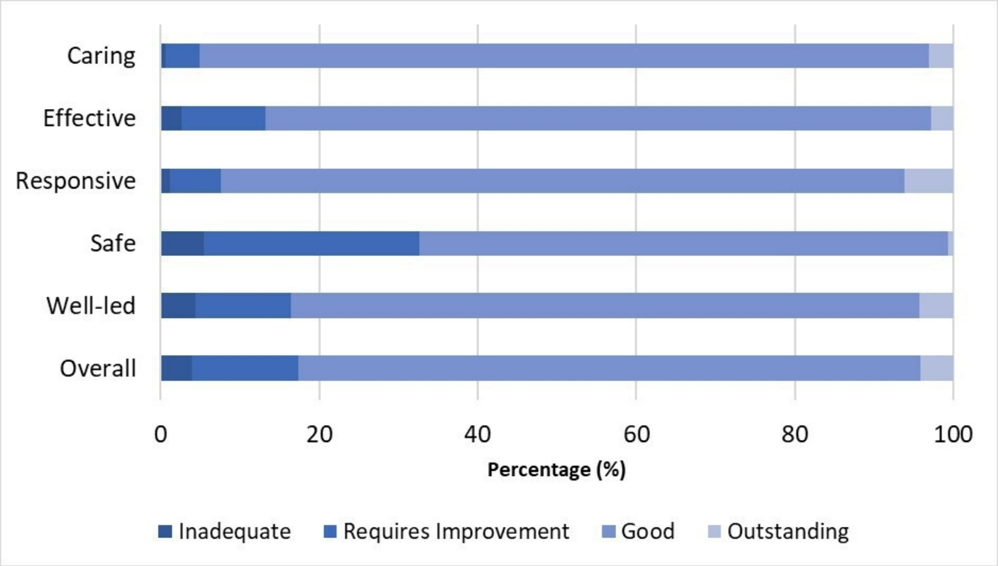

Figure 1 Distribution of CQC ratings by each domain

79x45mm (300 x 300 DPI)

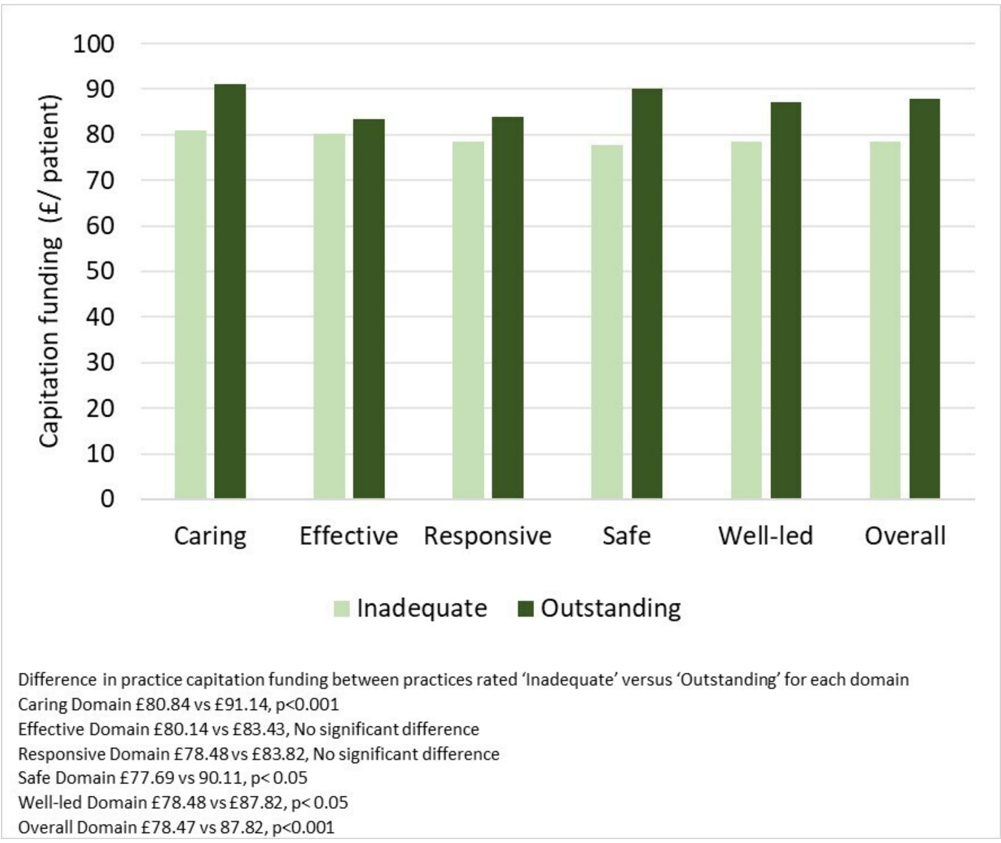

Figure 2 Practice capitation funding by overall practice rating 'Inadequate' versus 'Outstanding'

75x62mm (300 x 300 DPI)

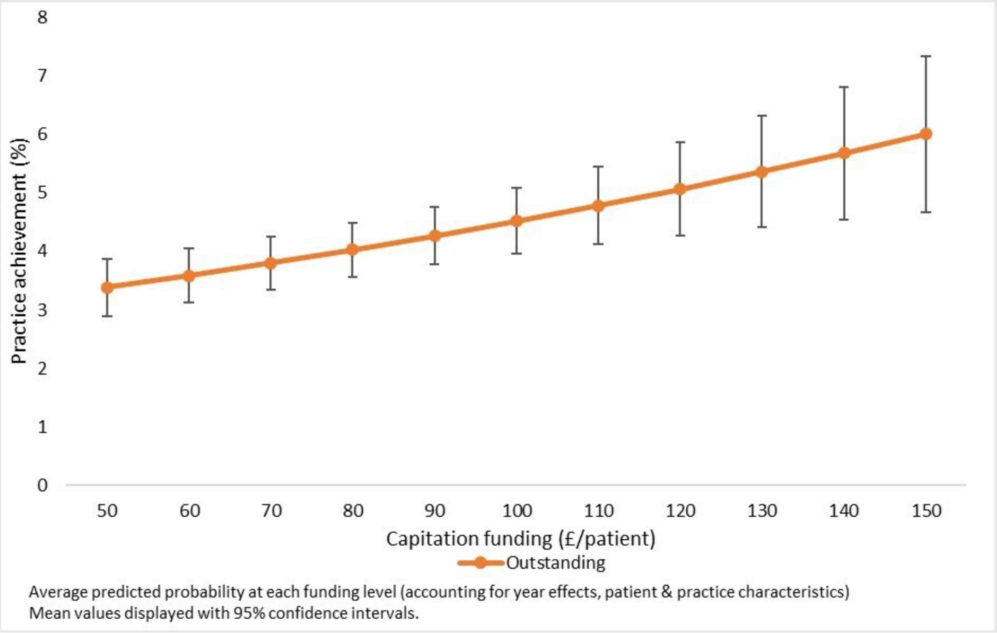

Figure 3 Estimated probability of overall practice rating of 'Outstanding' at various levels of capitation funding per registered patient

79x50mm (300 x 300 DPI)

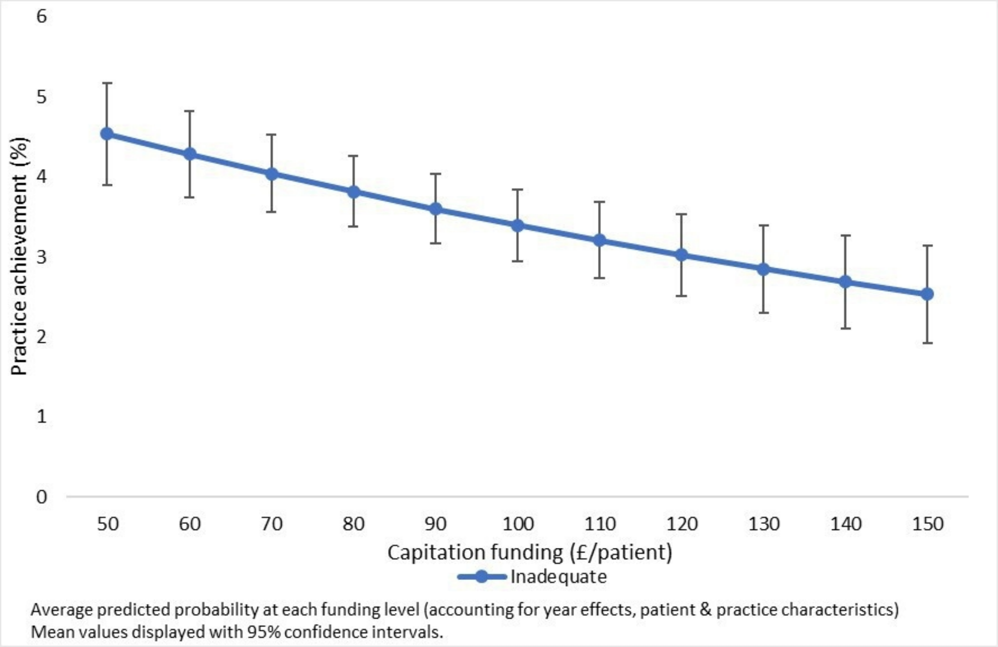

Figure 4 Estimated probability of overall practice rating of 'Inadequate' at various levels of capitation funding per registered patient

75x49mm (300 x 300 DPI)

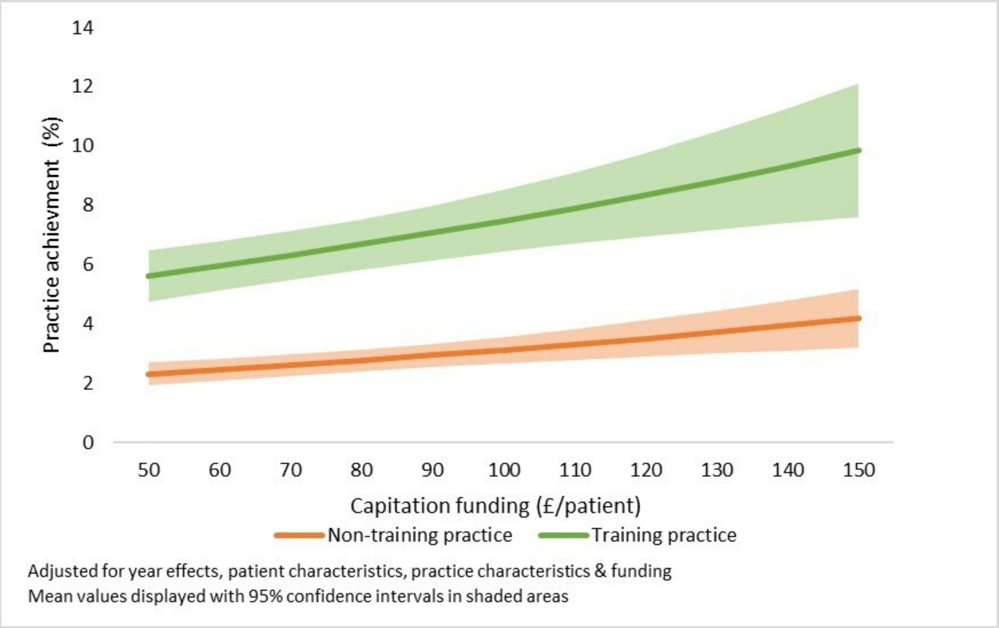

Figure 5 Estimated probability of 'Outstanding' overall practice CQC rating training versus non-training practices

73x46mm (300 x 300 DPI)

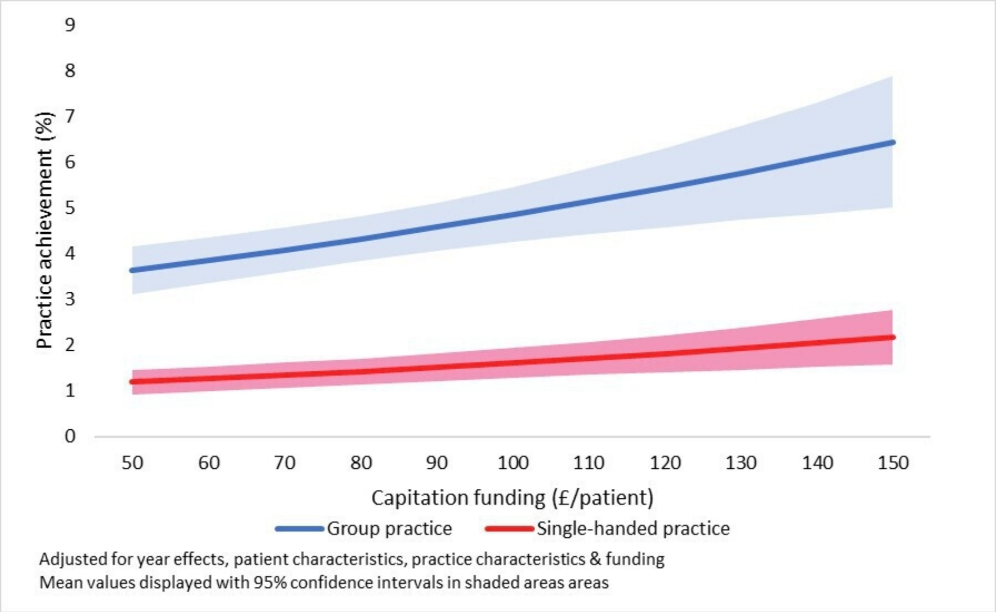

Figure 6 Estimated probability of 'Outstanding' overall practice CQC rating single-handed versus group practices

76x46mm (300 x 300 DPI)

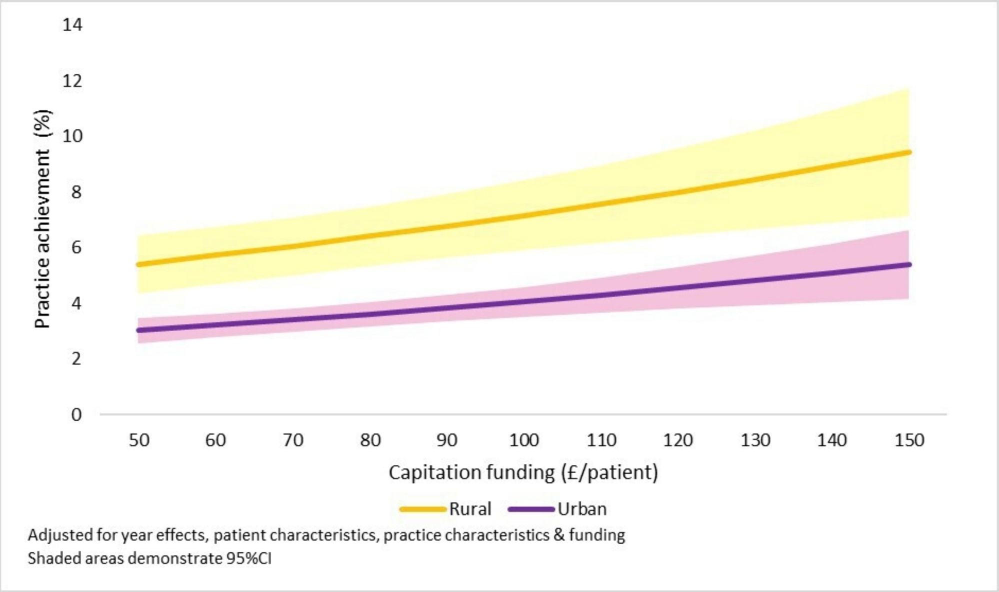

Figure 7 Estimated probability of ‘Outstanding’ overall practice CQC rating rural and urban practices

79x47mm (300 x 300 DPI)

STROBE Statement—checklist of items that should be included in reports of observational studies

	Item No.	Recommendation	Page No.	Relevant text from manuscript
**Title and abstract**	1	(*a*) Indicate the study's design with a commonly used term in the title or the abstract	1	"Cross-sectional study"
		(*b*) Provide in the abstract an informative and balanced summary of what was done and what was found	1	Summarised in Abstract: Design, Participants, Main outcome measures, Results
**Introduction**				
Background/rationale	2	Explain the scientific background and rationale for the investigation being reported	2-3	The link between measured dimensions of quality and primary care funding data
Objectives	3	State specific objectives, including any prespecified hypotheses	3	"we assess the relationship of practice capitation funding with overall CQC ratings and with the individual CQC domains"
**Methods**				
Study design	4	Present key elements of study design early in the paper	5	Presented under'Sample'.
Setting	5	Describe the setting, locations, and relevant dates, including periods of recruitment, exposure, follow-up, and data collection	3	Presented under 'Data Sources'
Participants	6	(*a*) *Cohort study*—Give the eligibility criteria, and the sources and methods of selection of participants. Describe methods of follow-up<br>*Case-control study*—Give the eligibility criteria, and the sources and methods of case ascertainment and control selection. Give the rationale for the choice of cases and controls<br>*Cross-sectional study*—Give the eligibility criteria, and the sources and methods of selection of participants	5	Cross-sectional study: general practice eligibility criteria summarised under 'Sample'
		(*b*) *Cohort study*—For matched studies, give matching criteria and number of exposed and unexposed<br>*Case-control study*—For matched studies, give matching criteria and the number of controls per case		

For peer review only - http://bmjopen.bmj.com/site/about/guidelines.xhtml

Variables	7	Clearly define all outcomes, exposures, predictors, potential confounders, and effect modifiers. Give diagnostic criteria, if applicable	4/5	Practice variables summarised under the heading 'practice data'; funding variables summarised under the heading 'practice funding data'
Data sources/ measurement	8*	For each variable of interest, give sources of data and details of methods of assessment (measurement). Describe comparability of assessment methods if there is more than one group	3	All sources of data listed under the heading, 'Data Sources'
Bias	9	Describe any efforts to address potential sources of bias	4	The large no. of variables included in our regression model are designed to maximise the explanatory power of the model
Study size	10	Explain how the study size was arrived at	5	Included practices represent ALL practices, minus exclusions based on predetermined exclusion criteria. Only lack of data availability restricted the sample size.

Continued on next page

For peer review only

Quantitative variables	11	Explain how quantitative variables were handled in the analyses. If applicable, describe which groupings were chosen and why	5/6	Quantitative variables analysed according to description in section headed, 'Data Analysis'.
Statistical methods	12	(*a*) Describe all statistical methods, including those used to control for confounding	5/6	Regression modelling – see Data Analysis
		(*b*) Describe any methods used to examine subgroups and interactions	6	Sub-groups such as 'outstanding' or 'inadequate' practices identified
		(*c*) Explain how missing data were addressed	5/6	Practices excluded if missing data
		(*d*) *Cohort study*—If applicable, explain how loss to follow-up was addressed<br>*Case-control study*—If applicable, explain how matching of cases and controls was addressed<br>*Cross-sectional study*—If applicable, describe analytical methods taking account of sampling strategy	n/a	The study was not a sample
		(*e*) Describe any sensitivity analyses	8	See heading 'Sensitivity analyses'
**Results**				
Participants	13*	(a) Report numbers of individuals at each stage of study—eg numbers potentially eligible, examined for eligibility, confirmed eligible, included in the study, completing follow-up, and analysed	5	Summarised under 'sample'. 7310 practices in England; 54 excluded.
		(b) Give reasons for non-participation at each stage	n/a	n/a (exclusion criteria stated above)
		(c) Consider use of a flow diagram	n/a	Exclusion criteria summarised on pg5 without use of a Flow diagram
Descriptive data	14*	(a) Give characteristics of study participants (eg demographic, clinical, social) and information on exposures and potential confounders	13	Table 2 summarises, 'Characteristics of general practices and their populations in England'
		(b) Indicate number of participants with missing data for each variable of interest	n/a	Sample only included those with available data
		(c) *Cohort study*—Summarise follow-up time (eg, average and total amount)	n/a	
Outcome data	15*	*Cohort study*—Report numbers of outcome events or summary measures over time	n/a	
		Case-control study—Report numbers in each exposure category, or summary measures of exposure	n/a	
		Cross-sectional study—Report numbers of outcome events or summary measures	13	Summary measures in Table 2
Main results	16	(*a*) Give unadjusted estimates and, if applicable, confounder-adjusted estimates and their precision (eg, 95% confidence interval). Make clear which confounders were adjusted for and why they were included	14	Table 4 summarises 'unadjusted' and three levels of 'adjusted' analyses

For peer review only - http://bmjopen.bmj.com/site/about/guidelines.xhtml

		15	Confounders adjusted for are listed in Table 5
	(*b*) Report category boundaries when continuous variables were categorized	n/a	No categorisation of continual variables
	(*c*) If relevant, consider translating estimates of relative risk into absolute risk for a meaningful time period	n/a	Absolute financial values are presented in the findings.

Continued on next page

For peer review only

For peer review only - http://bmjopen.bmj.com/site/about/guidelines.xhtml

Other analyses	17	Report other analyses done—eg analyses of subgroups and interactions, and sensitivity analyses	8	Sensitivity analyses summarised
**Discussion**				
Key results	18	Summarise key results with reference to study objectives	9	Key finding summarised at opening of Discussion: 'This study has demonstrated that higher capitation funding is associated with significantly higher overall practice quality ratings and ratings across all individual domains.'
Limitations	19	Discuss limitations of the study, taking into account sources of potential bias or imprecision. Discuss both direction and magnitude of any potential bias	10	Discussed under heading, 'Strengths and Weaknesses of the study'
Interpretation	20	Give a cautious overall interpretation of results considering objectives, limitations, multiplicity of analyses, results from similar studies, and other relevant evidence	10/11	Cautious interpretation summarised with reference to the literature
Generalisability	21	Discuss the generalisability (external validity) of the study results	11	Summarised under, 'Implications for policy and practice'
**Other information**				
Funding	22	Give the source of funding and the role of the funders for the present study and, if applicable, for the original study on which the present article is based	In submission details	First author is funded as Doctoral Research Fellow (DRF) by NIHR

*Give information separately for cases and controls in case-control studies and, if applicable, for exposed and unexposed groups in cohort and cross-sectional studies.

Note: An Explanation and Elaboration article discusses each checklist item and gives methodological background and published examples of transparent reporting. The STROBE checklist is best used in conjunction with this article (freely available on the Web sites of PLoS Medicine at http://www.plosmedicine.org/, Annals of Internal Medicine at http://www.annals.org/, and Epidemiology at http://www.epidem.com/). Information on the STROBE Initiative is available at www.strobe-statement.org.

For peer review only - http://bmjopen.bmj.com/site/about/guidelines.xhtml
